# Is the microbiome the answer to inflammatory bowel disease: systematic review

**DOI:** 10.1007/s00423-025-03897-0

**Published:** 2025-11-04

**Authors:** Devansh Shah, Fiona Phan, Zirong Yu, Joseph Do Woong Choi, James Wei Tatt Toh

**Affiliations:** 1 Department of Colorectal Surgery, Westmead Hospital, Corner Hawkesbury Road and Darcy Roads, Westmead, NSW 2145 Australia; 2 Department of Medicine, Liverpool Hospital, Corner Elizabeth Street and Goulburn Street, Liverpool, NSW Australia; 3 Department of Surgery, Princess Alexandra Hospital, Ipswich Road, Woolloongabba, QLD Australia; 4 Faculty of Medicine, Western Sydney University, Sydney, NSW Australia; 5 Faculty of Medicine and Health, The University of Sydney, Sydney, NSW Australia

**Keywords:** Microbiome, Dysbiosis, Inflammatory bowel disease

## Abstract

**Purpose:**

Inflammatory bowel disease (IBD) encompasses two main conditions - Crohn’s disease (CD) and ulcerative colitis (UC). Its pathogenesis is vastly unknown but genetics, environmental factors and the gut microbiome are thought to play vital roles. While dysbiosis is thought to be a feature of IBD, its exact role in pathogenesis is unclear.

**Methods:**

Relevant studies were identified through searching Medline and Embase from database inception to January 2025. Only gastrointestinal microbiome studies comparing IBD human patients with healthy controls (HC), performed on faecal, mucosal biopsy, saliva, or oral swab samples were examined. Studies were excluded if they included ≤ 10 IBD patients, did not compare IBD to HC, reported on IBD with other gastrointestinal infections, all were taking IBD medications, or included post-operative bowel resection patients.

**Results:**

Of 83 identified observational studies, most reported reduced alpha and beta diversity in IBD, more prevalent in CD than UC. There was depletion of protective butyrate producing *Firmicutes* bacteria including *Faecalibacterium* (specifically *F. prausnitzii*), *Eubacteria*, *Roseburia*, *Lachnospiraceae*, *Ruminococcaceae* (mainly *R. bromii*). There was decreased *Bacteroidetes* phylum in IBD, with depletion of Bacteroides genus in CD but increased in UC. There was increased Proteobacteria and its family Enterobacteriaceae in IBD.

**Conclusions:**

The gut microbiome in IBD demonstrated reduced biodiversity, more pronounced in CD, with increased pathogenic and reduced beneficial bacteria. While this study demonstrated important associations between the microbiome and IBD, the exact mechanism, whether it be from a multistep process, a causative agent, or interplay between mucosal immunology and dysbiosis, is yet be elucidated.

**Supplementary Information:**

The online version contains supplementary material available at 10.1007/s00423-025-03897-0.

## Introduction

Inflammatory bowel disease (IBD) is a disease spectrum characterised by inflammation and lesions primarily along the gastrointestinal tract (GIT), though patients may also have extraintestinal manifestations. There are two primary IBD subtypes, Crohn’s disease (CD) and ulcerative colitis (UC). 10% of patients however may not be initially differentiable and are referred to as IBD unclassified (IBD-U) [1, 2]. IBD is a disease of increasing incidence with a 0.06% prevalence worldwide and 0.25% prevalence in the USA in 2019 [3]. It is most prevalent in the North American and European countries compared to Africa and Asia, with marginally higher incidence of CD than UC and CD affecting females preferentially [1]. To this day, the cause of UC and CD has remained elusive, with genetics and the microbiome thought to play a major role.

The potential for the gut microbiome to cause human diseases is not new and dates back to the 1600s. The first human observation took place in the 1680 s at the hand of Dutch merchant and “father of microbiology” Antonie van Leeuwenhoek, when he examined the diversity between his faecal and oral specimens [4]. Louis Pasteur, a French chemist, and Robert Koch, a German physician helped gain acceptance of “Germ Theory of Disease”, developing the anthrax vaccine, and establishing its microbial cause by developing the pure culture technique respectively [5]. Henry Tissier, a French paediatrician, in 1899 was the first to isolate Bifidobacterium (Bacillus bifidus communis) from the faeces of breast fed infants to treat diarrhoea [6]. During 1917, in World War I, microbiologist Alfred Nissle discovered a strain of *E. coli* from the faeces of a German soldier [7]. In 1930, Dr Shirota created Yakult, a fermented milk drink, containing the beneficial strain Lactobacillus casei shirota [8]. These collective efforts have led to rapid growth in microbiome research more recently, including the Human Microbiome Project, launched in 2007 which collated and characterised the vast array of gut microbes [9]. Faecal microbiota transplant (FMT) has also been studied as a therapeutic, being the gold standard for treatment of recurrent or antibiotic-resistant *C. difficile* infection and *C. difficile* pseudomembranous colitis [10].

The microbiome has certainly received significant attention over the past few decades. However, while dysbiosis in IBD has generated significant interest, it has yet to translate into widespread clinical practice. Unlike the prominence of FMT in *C. difficile* management, its use in IBD treatment to re-establish good bacterial microbiota has not been as effective as one would hope. A recent 2023 Cochrane systematic review demonstrated that while FMT may induce clinical and endoscopic remission in active UC, the evidence was uncertain regarding effectiveness in inducing remission in CD or maintaining remission in UC or CD [11]. Moreover, some studies have reported worsening of IBD after FMT, with unknown long term safety data [12].

The gut microbiome is a complex ecosystem. The majority of intestinal microbes in healthy individuals is constituted by the phyla *Firmicutes*, *Bacteroidetes*, *Proteobacteria*, and *Actinobacteria* [13]. *Firmicutes* and *Bacteroidetes* together make up 90% of these phyla and are responsible for short-chain fatty acid (SCFA) production by fermenting ingested plant fibres. SCFAs, predominantly butyrate and propionate act as nutrients to promote healthy colonic epithelium and help regulate intestinal immune homeostasis [14].

In active IBD, the gut microbiome has been shown to display significant dysbiosis in diversity and composition compared to healthy controls. Whilst there can be variation between individuals and disease phenotypes, IBD in general has been characterised by a reduction in microbial diversity. Reduced *Firmicutes* and increased *Proteobacteria* (such as *Enterobacteriaceae*, *Bilophila*) and an increase in certain *Bacteroidetes* subclasses have previously been described [13]. Gut dysbiosis can lead to a loss of intestinal homeostasis and autoimmune activation with impaired intestinal epithelial tight junction integrity, potentially producing inflammation [13, 15].

Whilst the adult gut microbiome is generally stable, it may be altered by the use of antibiotics and probiotics, illness such as infective gastroenteritis or colitis from *Clostridium difficile*, primary sclerosing cholangitis, medications (including those taken for the treatment of IBD), and gastrointestinal surgery [16–18].

With the gut microbiome being such a complex ecosystem and its role in IBD still widely debated, the aim of this study was to update the current evidence by systematically reviewing the current literature. The study focuses on observational studies assessing differences in the microbiome between IBD and healthy patients, providing a better understanding of the role of dysbiosis in IBD, and the differences in the gut microbiome between UC and CD.

## Materials and methods

The systematic review was performed and reported in accordance with the Preferred Reporting Items for Systematic Reviews and Meta-Analyses (PRISMA) guidelines [19]. A review protocol was submitted in advance to PROSPERO, a database of systematic review protocols (registration ID: CRD42023466589).

### Search strategy

A comprehensive electronic search was performed using MEDLINE database via PubMed, and Embase (OvidSP) to identify published case-controlled articles on the gastrointestinal microbiome and inflammatory bowel disease (IBD) (Crohn’s disease and ulcerative colitis), from database inception to January 2025. In addition, the reference lists of the included studies were studied to identify further relevant studies. The relevant Medical Subject Headings (MeSH) terms used on MEDLINE were: (((“Inflammatory Bowel Diseases” OR “Crohn Disease” OR “Colitis, Ulcerative”) OR “Colitis”)) AND (“Gastrointestinal Microbiome” OR “Microbiota”) with limits on human subjects. On Embase, search terms used were: (exp inflammatory bowel disease/or exp Crohn disease/or exp ulcerative colitis/) AND ((exp microbiome/or exp bacterial microbiome/) OR (exp dysbiosis/or exp intestine flora/)), with subsequent limits set to human subjects, and MEDLINE duplicates removed.

### Study selection and patient population

The inclusion criteria were gastrointestinal microbiome studies comparing IBD human patients with healthy controls (HC), performed on faecal, intestinal mucosal biopsy, saliva, or oral swab samples. Both paediatric and adult patients were included. Subgroup analyses of studies assessing patients with CD or UC separately, active and remission disease separately, were included. Subgroup analyses focusing on only paediatric patients only, or treatment-naïve patients only, were included. Studies comparing the use of next generation DNA sequencing (NGS) compared to non-NGS, and assessing data based on sample type (faecal, mucosal biopsy, or oral) were all included. A study was excluded if it was not an observational study, included 10 or less IBD patients, was exclusively a fungal or virome study, or analysed different IBD subgroups only without comparison to healthy controls. Studies were also excluded if all patients in the study with IBD were on medical therapy. Studies including postoperative bowel resection patients, or IBD with other gastrointestinal infections (e.g. *Clostridium difficile*) were also excluded. The reason for excluding bowel resection patients or those with other gastrointestinal infections was their ability to alter the gut microbiome and confound results. IBD medications have a similar effect but due to most patients taking these, only those studies where all patients were taking IBD medications were excluded. Other results from the initial search that were excluded were studies not written in English with no available translation, with no full texts available, as well as conference proceedings, letters to the editor, or reviews.

### Outcomes

The primary outcome was to assess the difference in individual gastrointestinal bacterial species between IBD patients (CD and UC), and controls. The analysis of samples was performed using 16 S rRNA sequencing, DNA extraction, NGS, whole-genome shotgun sequencing, Fluorescent in situ hybridization (FISH), and flow cytometry. The secondary outcomes were to analyse the α (measuring diversity of bacteria isolated from a single sample) and β (examining the differences in bacteria between two samples) microbial diversity, and to tabulate any microbiome differences in UC, CD, active disease and remission, paediatric population, treatment-naïve population, sample type, and microbial analysis technique. The authors classified the primary and secondary outcomes into the following categories: significantly increased in IBD patients, significantly decreased in IBD patients, and no significant difference in IBD patients compared to controls.

### Eligibility assessment and data extraction

Articles were screened by full text, abstracts and titles by two authors (DS and FP). Both parties reviewed and scrutinized the relevance, eligibility and quality of the studies independently. Four authors (DS, FP, JC and ZY) independently reviewed the selected studies for complete analysis, and extracted data and entered it into a spreadsheet. One author (ZY) evaluated the accuracy of this process. When there was a discrepancy between reviewers, the data was rechecked, and an agreement reached by consensus by all authors. If the authors were unable to reach a consensus, the senior author (JT) arbitrated. The data collected included: IBD subtype (CD, UC), age group, number of patients, sex, IBD activity (remission or active), type of current treatment of IBD (aminosalicylates, steroids, immunomodulators, biologics), details of microbiota collection and evaluation (stool, mucosal biopsy, oral), microbiome analysis method, and α and β microbial diversity. Taxonomic classification differences were significant if P value < 0.05.

### Quality assessment

For eligible studies, two investigators (FP, DS) used the Newcastle-Ottawa Scale (NOS) independently to appraise the methodological quality over three domains, including the study/control population selection, comparability, and outcome evaluation. Any disagreements were resolved by arbitration by a third reviewer (JT). The standard 9-point scale was used for cohort and case-control studies, and a modified 10-point scale was used for cross-sectional studies [20, 21].

### Results

**Fig. 1:**
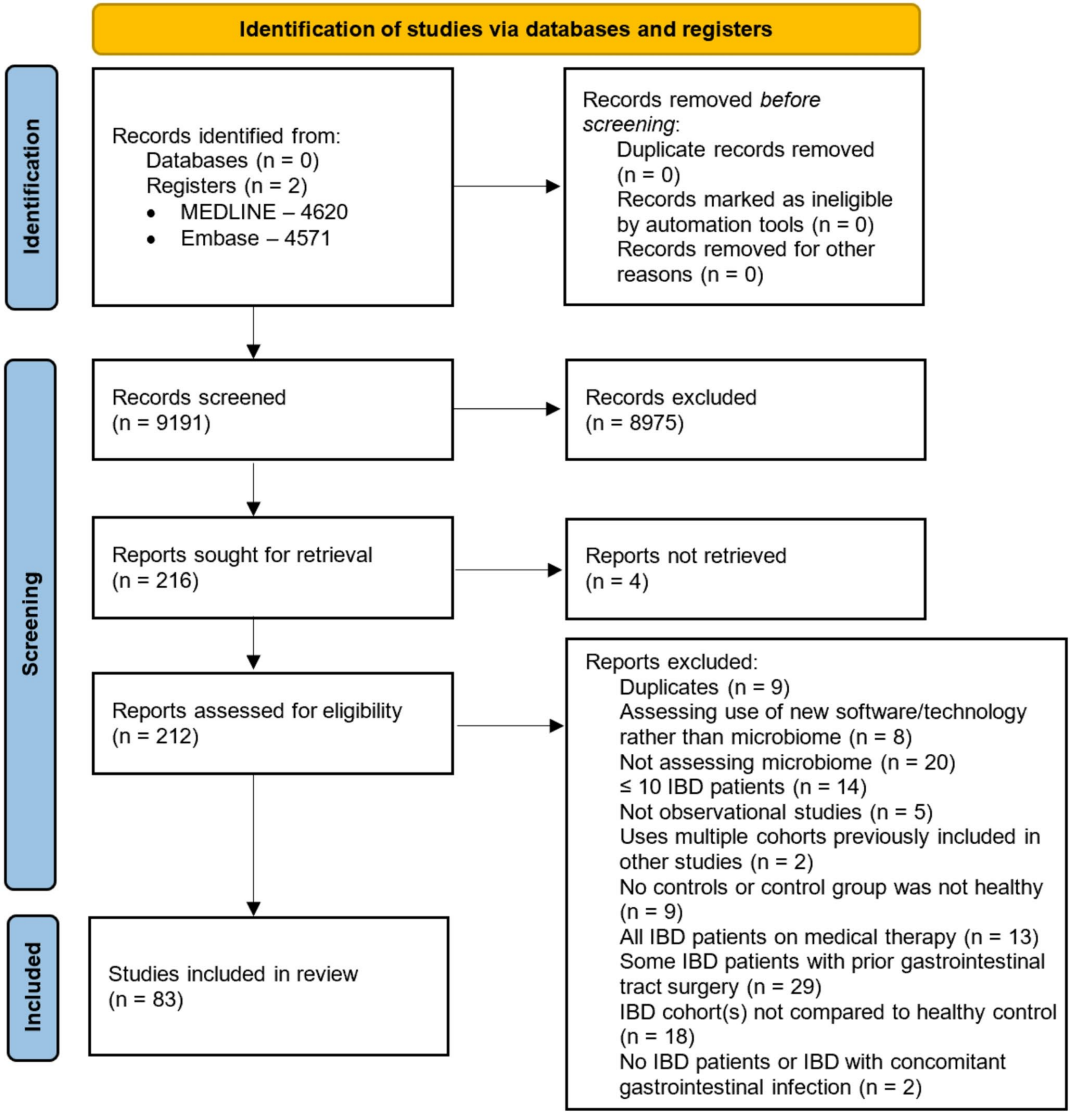
PRISMA flow diagram summarising the literature search and reviewing process

After applying inclusion/exclusion criteria, 83 studies were identified for inclusion from the original 4620 MEDLINE and 4571 Embase results. A PRISMA flow diagram summarising the selection process is seen in Fig. 1.

The data from these studies was tabulated in detail in Supplementary Table S1. Using the collected data, these studies were separated into various sub-categories, being studies assessing overall IBD vs. HC (*n* = 41), CD vs. HC (*n* = 51), UC vs. HC (*n* = 46), patients with active disease (*n* = 16) or in remission (*n* = 10) and comparing the two groups (*n* = 7). Other subgroups included paediatric only studies (*n* = 20), treatment-naïve only studies (*n* = 8), use of NGS (*n* = 75) vs. non-NGS (*n* = 12), and results based on faecal (*n* = 65) vs. mucosa biopsy or brushing (*n* = 19) vs. oral microbiome (*n* = 10) samples. Studies primarily consisted of cross-sectional studies with 57 of the 83 studies, followed by cohort studies (15), and finally case-control studies (11).

### Quality assessment

Use of the NOS found that the majority of studies were generally of a low risk of bias [20, 21]. Regarding the cross-sectional studies which constituted the majority of literature analysed in our review, studies primarily were not awarded points if they did not provide calculations for power to support the adequacy of their sample sizes. Points were also not awarded in the NOS across cohort, case-control, and cross-sectional studies where data was not available on age and gender to assess comparability of IBD and control groups. Of note, all cohort studies did not provide sufficient information to reflect their process of follow up. With regards to the included cohort studies and case-control studies, points were also not awarded in the domains of selection of non-exposed cohorts or controls. This is summarised in Supplementary Tables S2-4. As such, all studies were of either high (64) or moderate (19) quality of evidence based on the NOS scores. Cohort and case control studies were of high quality with 7 or more stars, moderate quality with 4–6 stars, and low quality with 3 or less stars. Cross sectional studies were high quality with 8 or more stars, moderate quality with 5–7 stars, and low quality with 4 or less stars.

### Microbial diversity

Diversity was subcategorised into alpha, within one sample, and beta, between samples. Most studies reported a significant reduction in alpha diversity in IBD or phenotype (UC/CD) compared to HC and similarly, a significant difference in beta diversity between IBD and HC, occasionally distinguishing between UC and CD (Table 1). However, in 12 studies both alpha and beta diversity were not assessed, in one study alpha diversity was not assessed, and in a further two, beta diversity was not assessed.

**Table 1:** Summary of included study findings. FISH – fluorescence in situ hybridization; qPCR – quantitative PCR; IBD – inflammatory bowel disease; CD – Crohn’s disease; UC – ulcerative colitis; HC – healthy control; HS – healthy sibling; metagenomic shotgun sequencing – MSS; 16 S rRNA GS – 16 S rRNA gene sequencing; m-ddPCR - multiplexed droplet digital PCR; N/S – Not specified

Author	Year	Study Design	Participants	Disease Activity	Treatment	Specimen	Analysis Method	Microbial (alpha/beta) Diversity
Budzinskia et al. [38]	2025	Cross-sectional study	CD Adult	N/S	N/S	Faeces	16 S rRNA GS	*Beta* different CD vs. HC
Zheng et al. [39]	2024	Cross-sectional study	CD/UC Adult	N/S	Some	Faeces	m-ddPCR	*Alpha* reduced in CD/UC vs. HC. No difference CD vs. UC *Beta* different UC > CD vs. HC
Chen et al. [40]	2024	Cross-sectional study	CD Adult	52% active	Some	Faeces	16 S rRNA GS	*Alpha* different between CD vs. HC. No difference in active vs. remission CD *Beta* different CD vs. HC. No difference in active vs. remission CD
Conrad et al. [41]	2024	Cross-sectional study	CD/UC Paediatric	46% active	Some	Faeces	Metagenomic shotgun sequencing	*Alpha* reduced in IBD vs. HC *Beta* different between IBD vs. HC
Scanu et al. [42]	2024	Case-control study	UC Adult	N/S	Some	Faeces	16 S rRNA GS	*Alpha* not different in UC vs. HC *Beta* different UC vs. HC
Alahdal et al. [43]	2024	Cohort study	CD Adult	31% active	N/S	Faeces	16 S rDNA GS	*Alpha* reduced in CD vs. HC *Beta* different CD vs. HC
Han et al. [44]	2024	Case-control study	CD/UC Adult	N/S	N/S	Faeces, saliva	16 S rRNA GS	*Alpha* reduced in IBD vs. HC *Beta* different IBD vs. HC
Alsulaiman et al. [45]	2023	Cross-sectional study	CD/UC Adult	N/S	N/S	Faeces	16 S rRNA GS	*Alpha* reduced in IBD vs. HC. *Beta* no difference IBD vs. HC (when adjusted for age and sex)
Markelova et al. [46]	2023	Cross-sectional	CD Adult	68.8% active	Some	Faeces	16 S rRNA GS	*Alpha* reduced in CD vs. HC *Beta* different CD vs. HC
Lopez et al. [47]	2023	Cross-sectional	IBD Paediatric	Unknown	Some	Faeces	16 S rRNA GS	*Alpha* reduced in IBD vs. HC *Beta* different IBD vs. HC
Elmaghrawy et al. [48]	2023	Cohort	CD/UC/UIBD Paediatric	93% active	None – all naïve	Tongue/buccal swab	16 S rRNA GS	*Alpha* reduced in CD vs. HC *Beta* different CD vs. HC
Wu et al. [49]	2023	Cross-sectional	CD Paediatric	58% active	Some	Faeces, mucosal biopsies	16 S rRNA GS	*Alpha* not reduced in CD vs. HC *Beta* not different CD vs. HC
Al-Amrah et al. [50]	2023	Cross-sectional	CD/UC Adult	72% active	Some	Faeces	16 S rRNA GS & qPCR	*Alpha* reduced in IBD vs. HC *Beta* different IBD vs. HC
Räisänen et al. [51]	2023	Case-control	IBD Paediatric	Unknown	N/S	Saliva	16 S rRNA GS	*Alpha* reduced in IBD vs. HC *Beta* different IBD vs. HC
Gao et al. [52]	2023	Cross-sectional	CD Adult	100% active	N/S	Faeces	16 S rRNA GS	*Alpha* not reduced in CD vs. HC *Beta* not different CD vs. HC
Salimi et al. [53]	2022	Cross-sectional	CD/UC Adult	33% active	N/S	Faeces	16 S rRNA GS	Number and diversity of microbiota reduced in IBD vs. HC Alpha/Beta diversity not described
Zhu et al. [54]	2022	Cross-sectional	UC Adult	50% active	N/S	Faeces	16 S rRNA GS	*Alpha* reduced in remission UC vs. HC (no difference inactive UC) *Beta* different UC vs. HC
Xu et al. [55]	2022	Cross-sectional	UC Adult	0% active All remission	N/S	Faeces	16 S rRNA GS	*Alpha* reduced in UC vs. HC *Beta* different UC vs. HC
Hu et al. [56]	2022	Cross-sectional	CD Adult	100% active	Some	Faeces	16 S rRNA GS	*Alpha* reduced CD vs. HC *Beta* (by PCoA) different CD vs. HC
Ma et al. [30]	2022	Cross-sectional	CD Adult	N/S	None	Faeces	16 S rRNA GS	*Alpha* reduced CD vs. HC *Beta* different CD vs. HC
Jacobs et al. [57]	2022	Cross-sectional	CD Adult	0% active All remission	Some	Faeces	16 S rRNA GS	*Alpha* reduced in CD vs. HC *Beta* different CD vs. HC
Sternes et al. [58]	2022	Cross-sectional	IBD Adult	N/S	Some	Faeces, mucosal biopsies	16 S rRNA GS	*Alpha* reduced in IBD vs. HC *Beta* different between IBD vs. HC
Paljetak et al. [59]	2022	Cross-sectional	CD/UC Adult	95% active	None – all naïve	Faeces, mucosal biopsies	16 S rRNA GS	Not assessed for IBD vs. HC
Hu et al. [60]	2022	Cross-sectional	CD Adult	N/S	Some	Saliva	16 S rRNA GS	*Alpha* reduced in CD vs. HC *Beta* different CD vs. HC
Zuo et al. [61]	2022	Cross-sectional	UC Paediatric	100% active	Some	Faeces	16 S rRNA GS & MSS	*Alpha* reduced in UC vs. HC *Beta* different UC vs. HC
Park et al. [62]	2022	Cohort	CD/UC Adult	N/S	Some	Faeces, saliva, serum, urine	16 S rRNA GS (faeces/saliva), Extracellular vesicles (faeces/saliva/serum/urine)	*Alpha* **faeces** - reduced in IBD vs. HC *Beta* **faeces** - no difference IBD vs. HC
Berbisá et al. [22]	2022	Cross-sectional	UC Adult	Active 42%	Some	Faeces	16 S rRNA GS	*Alpha* not reduced in UC vs. HC *Beta* not different UC vs. HC
Barberio et al. [63]	2022	Cross-sectional	UC Adult	43% active	Some	Faeces	16 S rRNA GS	*Alpha* reduced in active > remission UC vs. HC *Beta* different active UC vs. remission UC vs. HC
Sukhina et al. [64]	2022	Cross-sectional	IBD; Adult	N/S	N/S	Faeces	16 S rRNA GS, MSS	Not assessed
Teofani et al. [65]	2022	Cross-sectional	CD/UC Adult	32% active	N/S	Faeces	16 S rRNA GS	*Alpha* reduced in IBD vs. HC *Beta* different IBD vs. HC
Wan et al. [66]	2022	Cross-sectional	UC Adult	N/S	N/S.	Faeces	16 S rRNA GS	*Alpha* reduced in UC vs. HC *Beta* no difference UC vs. HC
Wang et al. [67]	2022	Case-control	IBD Adult	N/S	N/S	Faeces	16 S rRNA GS	*Alpha* reduced in IBD vs. HC *Beta* different IBD vs. HC
Hu et al. [68]	2021	Case-control	CD Adult	40% Active	Some	Faeces, saliva	MSS	*Alpha* **Faeces + Saliva** not reduced CD vs. HC *Beta* **Faeces** different CD vs. HC *Beta* **Saliva** not different CD vs. HC
Tang et al. [69]	2021	Case-control	UC Adult	100% active	None	Faeces	16 S rRNA GS	*Alpha* reduced in UC vs. HC *Beta* different UC vs. HC
Ostrowski et al. [70]	2021	Cross-sectional	CD Adult	N/S	Some	Mucosal biopsy, gastric fluid	16 S rRNA GS	*Alpha* not reduced CD vs. HC *Beta* different CD vs. HC
Liu et al. [71]	2021	Cross-sectional	UC Adult	100% active	N/S	Faeces	16 S rRNA GS	*Alpha* reduced in Uyghur UC vs. Uyghur HC - not reduced Han UC vs. Han HC *Beta* different Ughur (UC v HC) + Han (UC v HC)
Frau et al. [72]	2021	Cohort	CD/UC Adult	66% active	Some	Faeces, mucosal biopsies	16 S rRNA GS	*Alpha* F**aeces + Mucosa** reduced in CD vs. HC - no change in UC vs. HC *Beta* different CD vs. UC vs. HC
Liang et al. [73]	2021	Cross-sectional	IBD Adult	N/S	N/S	Faeces	MSS	*Alpha* reduced in IBD vs. HC *Beta* different IBD vs. HC
Nishihara et al. [74]	2021	Cohort	UC Adult	52% active	Some	Mucosal biopsies	16 S rRNA GS	*Alpha* reduced in UC vs. HC *Beta* different UC vs. HC
Xia et al. [75]	2021	Cross-sectional	CD/UC Adult	N/S	N/S	Faeces	MSS	*Alpha* reduced in IBD, especially CD vs. HC - reduced in UC (American cohort) vs. HC *Beta* different CD > UC vs. HC
Maldonado-Arriaga et al. [76]	2021	Cross-sectional	UC Adult	50% active	Some.	Faeces	16 S rRNA GS	*Alpha* reduced active UC vs. remission UC/HC *Beta* different active UC vs. remission UC/HC
Dai et al. [77]	2021	Cross-sectional	UC Adult	N/S	N/S	Faeces	16 S rRNA GS	*Alpha* reduced in UC vs. HC *Beta* different UC vs. HC
Chang et al. [78]	2021	Cross-sectional	CD/UC Adult	30% active	Some	Faeces	16 S rRNA GS	*Alpha* reduced in IBD and CD > UC vs. HC *Beta* different IBD vs. HC, CD vs. HC, UC vs. HC
Abdul-Hussein et al. [79]	2021	Cross-sectional	IBD Adult	N/S	N/S	Faeces	Culture	Not assessed
Juyal et al. [80]	2021	Cross-sectional	UC Adult	52.3% active	N/S	Faeces	16 S rRNA GS	*Alpha* reduced in UC vs. HC *Beta* different UC vs. HC
Sanchis-Artero et al. [81]	2021	Cohort	CD Adult	N/S	Some	Faeces	16 S rRNA GS	*Alpha* reduced in CD vs. HC *Beta* different CD vs. HC
Somineni et al. [82]	2021	Cohort	CD/UC Paediatric	45% active	Some	Faeces, saliva	16 S rRNA GS	*Alpha* reduced in IBD vs. HC *Beta* different IBD vs. HC
Alam et al. [83]	2020	Cross-sectional	CD/UC Adult	N/S	N/S	Faeces	16 S rRNA GS	*Alpha* reduced in CD vs. UC/HC. *Beta* not assessed
Sila et al. [84]	2020	Cross-sectional	CD/UC Paediatric	100% active	None – all naïve	Faeces	16 S rRNA GS	*Alpha* reduced in IBD vs. HS/HC *Beta* not assessed
Qiu et al. [85]	2020	Cross-sectional	CD Adult	76% active	Some	Faeces	16 S rRNA GS	*Alpha* reduced in CD vs. HC *Beta* not assessed
Kowalska-Duplaga et al. [86]	2019	Cross-sectional	CD Paediatric	100% active	None – all naïve	Faeces	16 S rRNA GS	*Alpha* reduced in CD vs. HC *Beta* different in CD vs. HC
Guo et al. [87]	2019	Cross-sectional	CD/UC Adult	71.6% active	None	Faeces	qPCR	Not assessed
Malham et al. [88]	2019	Cross-sectional	CD/UC/UIBD Paediatric	N/S	Some	Faeces	16 S rRNA GS	*Alpha* reduced in IBD vs. HC *Beta* not different IBD/CD/UC vs. HC
Olbjørn et al. [89]	2019	Case-control	CD/UC/UIBD Paediatric	N/S	None – all naïve	Faeces	16 S rRNA GS	Not assessed
Kansal et al. [90]	2019	Cohort	CD Paediatric	91.3% active	Some	Mucosal biopsies	16 S rRNA GS	*Alpha* reduced in CD vs. HC *Beta* different first diagnosis CD vs. HC - significantly different between first diagnosis/relapse/remission CD
Zhong et al. [91]	2019	Cross-sectional	UC; Adult	57.1% active	Some	Mucosal biopsy	FISH	Not assessed
Al-Bayati et al. [92]	2018	Cross-sectional	UC; Adult	N/S	N/S	Mucosal biopsies	16 S rRNA GS	Not assessed
Xun et al. [93]	2018	Case-control	CD/UC Adult	32.8% active	Some	Saliva	16 S rRNA GS	*Alpha*: not different UC/CD/HC *Beta*: different UC vs. CD vs. HC
de Meij et al. [94]	2018	Cohort	CD/UC Paediatric	100% active	None – all naïve	Faeces	qPCR	*Alpha* not different IBD vs. HC *Beta* different IBD vs. HC
Walujkar et al. [95]	2018	Cohort	UC Adult	100% active	None	Mucosal biopsies	16 S rRNA GS, qPCR	*Alpha* reduced in UC vs. HC *Beta* different UC vs. HC
Ma et al. [96]	2018	Cross-sectional	CD/UC Adult	86.2% active	Some	Faeces	16 S rRNA GS	*Alpha* reduced (Chao1 but not observed species/ACE) in IBD vs. HC *Beta* different IBD vs. HC + active CD/UC vs. HC
Nishino et al. [97]	2018	Cross-sectional	CD/UC Adult	42.0% active	Some	Mucosal brush samples	16 S rRNA GS	*Alpha* reduced significantly in IBD vs. HC, no significant differences between CD vs. UC *Beta* different CD > UC vs. HC
Imhann et al. [98]	2018	Cross-sectional	CD/UC/UIBD Adult	25.8% active	Some	Faeces	16 S rRNA GS	*Alpha* reduced in IBD vs. HC *Beta* different IBD vs. HC
Bajer et al. [99]	2017	Cross-sectional	UC Adult	N/S	Some	Faeces	16 S rRNA GS	*Alpha* reduced in UC vs. HC *Beta* different UC vs. HC
Knoll et al. [100]	2017	Case-control	CD/UC	66.7% active	Some	Faeces	MSS	*Alpha* reduced in UC (Shannon index) vs. HC - reduced in IBD overall (richness) vs. HC *Beta* not assessed
Sokol et al. [101]	2017	Cross-sectional	CD/UC Adult	45.1% active	Some	Faeces	16 S rRNA GS	*Alpha* reduced in IBD vs. HC *Beta* different CD vs. UC vs. HC
Rehman et al. [102]	2016	Cross-sectional	CD/UC Adult	0% active All remission	Some	Mucosal biopsies	16 S rRNA GS	*Alpha* reduced in CD > UC vs. HC *Beta* different IBD vs. HC
Yao et al. [103]	2016	Case-control	UC Adult	53.3% active	None	Faeces	qPCR	Not assessed
Hoarau et al. [104]	2016	Cross-sectional	CD Adult	15% active	N/S	Faeces	16 S rRNA GS	*Alpha* richness increased in CD + non-CD relatives vs. non-related controls *Beta* different CD vs. non-CD relatives vs. non-related controls
Shaw et al. [105]	2016	Cohort	CD/UC Paediatric	N/S	None – all naïve	Faeces	16 S rRNA GS	*Alpha* reduced in IBD vs. HC *Beta* different IBD vs. HC
Eun et al. [106]	2016	Cross-sectional	CD Adult	42.9% active	Some	Faeces, mucosal biopsy	16 S rRNA GS	*Alpha* reduced in CD vs. HC (for faeces but not mucosa) *Beta* different in CD vs. HC (for faeces but not mucosa)
Naftali et al. [107]	2016	Cross-sectional	CD Adult	35.5% active	Some	Mucosal biopsies	16 S rRNA GS	*Alpha* no difference in ileal vs. colonic CD, but generally reduced in IBD vs. HC *Beta* different ileal vs. colonic CD vs. HC
Kolho et al. [108]	2015	Cohort	CD/UC/UIBD Paediatric	30.9% active	Some	Faeces	Phylogenetic microarray, qPCR	*Alpha* reduced in IBD vs. HC *Beta* different IBD vs. HC before TNFa
Quince et al. [109]	2015	Cohort	CD Paediatric	100% active	Some	Faeces	16 S rRNA GS, MSS	*Alpha* reduced in CD vs. HC *Beta* different CD vs. HC [Before EEN]
Maukonen et al. [110]	2015	Cohort	CD/UC Paediatric	N/S	Some	Faeces	Culture, qPCR	Not assessed
Kabeerdoss et al. [111]	2015	Cross-sectional	CD/UC Adult	N/S	Some	Mucosal biopsy	16 S rRNA GS, qPCR	Not assessed
Chen et al. [112]	2014	Cross-sectional	CD/UC Adult	83% active	N/S	Faeces, mucosal biopsies	16 S rRNA GS	*Alpha* reduced in IBD vs. HC *Beta* different IBD vs. HC, not different CD vs. UC
Gevers et al. [113]	2014	Cohort	CD Paediatric	N/S	Some	Faeces, mucosal biopsies	16 S rRNA GS	Not assessed
Said et al. [114]	2014	Cross-sectional	CD/UC Adult	54% active	Some	Saliva	16 S rRNA GS	*Alpha* no difference in IBD vs. HC *Beta* different IBD (CD/UC) vs. HC
Tong et al. [115]	2013	Cross-sectional	CD/UC Adult	N/S	N/S	Muosal biopsies	16 S rRNA GS	*Alpha* reduced in IBD (CD > UC) vs. HC *Beta* different IBD (CD > UC) vs. HC
Prideaux et al. [116]	2013	Cross-sectional	CD/UC Adult	N/S	Some	Mucosal biopsies	16 S rRNA GS	*Alpha* reduced in CD vs. HC *Beta* different IBD vs. HC
Pérez-Brocal et al. [117]	2013	Cross-sectional	CD Adult	90% active	Some	Faeces	16 S rRNA GS	*Alpha* reduced in CD vs. HC *Beta* different CD vs. HC
Docktor et al. [23]	2012	Cross-sectional	CD/UC Paediatric	32% active	Some	Tongue/buccal swab	16 S rRNA GS	*Alpha* reduced in CD vs. HC - not reduced in UC vs. HC *Beta* not different CD vs. HC

Interestingly in a cross-sectional study of patients with UC in the Faroe Islands where there is the world’s highest prevalence of IBD, no differences were found in alpha or beta diversity between patients with UC and HC [22]. Notably, samples were from faeces analysed using NGS with a 3-month period prior to collection of no antibiotics. However, 93% of patients were on IBD treatment, including some on biological therapy.

In eight of the included studies, alpha and/or beta diversity was generally more significantly reduced in CD than UC. This change was consistent between both faecal and mucosal biopsy samples.

Four adult oral microbiome studies were found, where there was no difference in alpha diversity between IBD or phenotype compared to HC, though there was a significant difference in beta diversity. A further paediatric oral microbiome study found a significant difference in alpha diversity between CD and HC but not UC and HC, with no difference in beta diversity [23].

From five (of 20) paediatric studies, two had no significant difference in alpha diversity, though there was a difference in beta diversity between IBD and HC. These studies happened to be treatment-naïve. From the remaining studies, two had reduction in alpha diversity but no difference in beta diversity, with the remaining study having no difference in alpha or beta diversity.

#### Taxonomic profiles

Comparative levels of microbes in the gastrointestinal tract are compared at the taxonomic levels of phylum, family, and genus/species in Supplementary Table S1. Based on the analytic technique, each study did not always assess for the same microbes. The microbes included are those that were found to be significantly increased or decreased compared to HC.

### Phylum level

The major **phyla** noted amongst the studies were *Bacteroidetes*,* Firmicutes*,* Actinobacteria*,* Fusobacteria*,* Proteobacteria*,* and Verrucomicrobia*. *Firmicutes* was significantly depleted in IBD overall compared to HC (11 studies). This was more notable in CD with 9 studies finding a reduction compared to 4 UC studies. *Bacteroidetes* was noted be more commonly decreased in CD (8 studies with decrease) than UC (2 studies) compared to HC. *Fusobacteria* was noted to be increased in CD (5 studies) compared to HC. *Proteobacteria* (also called *Pseudomonadata*) was increased in 13 IBD studies compared to HC, more notably in CD (10 studies) than UC (4 studies).

On the other hand, there was a trend towards *Verrucomicrobia* depletion in IBD paediatric and treatment-naïve groups compared to HC, with 2 studies each demonstrating this.

*Proteobacteria* was increased and *Firmicutes* decreased in active IBD compared to remission with 2 studies showing this in UC and 1 in CD.

### Family level

At the **family** level, *Enterobacteriaceae* (*Proteobacteria* phylum) was increased in IBD compared to HC, but more pronounced in CD.

*Lachnospiraceae* (5 studies) and *Ruminococcaceae* (8 studies), both of *Firmicutes* phylum, were decreased in IBD compared to HC, with *Ruminococcaceae* being more predominantly depleted in CD (6 studies) than UC (4 studies). While overall *Firmicutes* levels were depleted in IBD, *Streptococcaceae* (3 studies) and *Veillonellaceae* (2 studies), both of *Firmicutes* phylum, were increased in IBD (both CD and UC) compared to HC.

### Genus/species level

Many microbes were present at the **genus/species** level. On assessment of their levels in various studies and subgroups, a few trends were apparent.

From the phylum *Firmicutes*, *Faecalibacterium* in 8 studies (particularly *Faecalibacterium prausnitzii*), *Roseburia* (8 studies), *Clostridium* (4 studies), and *Coprococcus* (5 studies) were depleted in IBD. This was the case more so in CD than UC compared to HC. On the other hand, *Dialister* (3 studies) and *Lachnospira* (2 studies) were more depleted in UC than CD. *Lactobacillus* (2 studies) was depleted in active UC compared to HC.

Also belonging to the phylum *Firmicutes*, *Ruminococcus* genus was depleted in IBD/CD/UC (23 studies) compared to HC with the common species being *R. bromii*. Depletion was more notable in active IBD (4 studies) and paediatric (5 studies) IBD. However, where *R. gnavus* was identified, it was enriched in IBD/CD/UC (9 studies) compared to HC.

Not all genera from the *Firmicutes* phylum were depleted. There were increased levels of *Enterococcus* (4 studies) in IBD. There were increased levels of *Streptococcus* in UC/IBD (5 and 6 studies respectively) but not in CD. *Veillonella* genus was increased in CD (5 studies) compared to HC.

Of the *Bacteroidetes* phylum, *Bacteroides* was depleted in CD (7 studies) compared to HC but enriched in UC (11 studies) compared to HC. No difference in *Bacteroides* was seen in IBD overall. Similarly, *Prevotella* was reduced in CD (4 studies), with this most evident in active CD (2 studies) compared to HC, with no change in UC.

Of the *Proteobacteria* phylum, *Escherichia* and/or *Shigella* genera (11 studies), Klebsiella (4 studies), and *Haemophilus* (5 studies) were increased in IBD/CD/UC compared to HC. The *Escherichia* and/or *Shigella* genera was increased (31 studies) particularly in the active CD subgroup (4 studies).

*Bifidobacterium* (*Actinomycetota* phylum) was increased in UC (4 studies) compared to HC but depleted in CD (3 studies) compared to HC. *Fusobacterium* (*Fusobacteria* phylum) was increased in IBD/CD/UC (9 studies) compared to HC.

### Oral vs. mucosal vs. faecal samples

Results from oral samples differed from mucosal and faecal samples. Mucosal biopsy and faecal samples demonstrated increased *Proteobacteria* (3 mucosal, 11 faecal) and decreased *Firmicutes* (2 mucosal, 9 faecal studies) in IBD. Interestingly, in oral samples *Proteobacteria* was noted to be depleted in IBD (3 studies) compared to HC. *Firmicutes* was also depleted in oral IBD and CD samples in 1 study but in another study, increased in UC. In oral samples, *Fusobacteria* was also noted to be depleted in IBD, however this was only noted in 1 study which involved paediatric, treatment-naïve patients only. Conversely, there was an increase in IBD noted in mucosal/faecal samples (7 studies). In contrast, *Veillonella* was increased in CD consistently between faecal CD (4 studies), faecal UC (3 studies), mucosal UC (2 studies), and oral UC (2 studies) samples.

## Discussion

This study confirms findings of prior systematic reviews on the IBD microbiome while providing an updated inclusion of the latest evidence. Through employing a comprehensive inclusion and exclusion criteria, the authors were able to minimise many potential confounders such as prior intestinal surgery, significant IBD medication use, and concomitant gastrointestinal disease which can alter the IBD microbiome [16–18]. Overall, 83 studies were identified, and further divided into subgroups based on cohort characteristics and the IBD subtypes studied.

### Diversity

As established in the literature, alpha diversity is typically reduced in IBD compared to control patients and the findings of this study were concurrent, with 61 of the 83 studies demonstrating reduced alpha diversity between IBD or phenotype (CD/UC) compared to HC [24]. Of the studies that did not demonstrate this, 13 did not assess alpha diversity, four were oral microbiome studies with no difference in alpha diversity, three were paediatric studies of which two were treatment-naïve, and one constituted of a UC population from a unique Northern European Island population. Similarly, there are established differences in beta diversity evidenced in the literature between IBD compared to control cohorts, that was also reflected in our study. 63 of the 83 studies concluded there was a significant difference in beta diversity between IBD or phenotype compared to HC [25]. Fourteen of the studies that did not support this finding did not assess beta diversity. In eight studies, alpha and/or beta diversity was more significantly reduced/altered in CD than UC compared to HC, which is supported in previous literature, particularly with regards to more significant loss of alpha diversity in CD compared to UC [25].

### Taxonomic analysis

In analysing the taxonomic profile of the IBD microbiome, more information was provided into the interdependent relationship between dysbiosis, dysregulation of homeostatic GIT function, and the autoimmune processes involved in the pathogenesis of the disease. Our findings of bacterial dysbiosis at the phylum, family, and genus/species levels are supported by the existing literature.

The finding in our study of reduced levels of the phylum ***Firmicutes*** is echoed in multiple prior studies, with bacteria within this group playing a protective role within the intestinal lumen [26]. The protective effect from *Firmicutes* results from many of its bacteria being butyrate (a type of SCFA) producers, useful for providing intestinal epithelial nutrition, and thus maintaining immune homeostasis [14]. Within this phylum, *Faecalibacterium* (specifically *F. prausnitzii*), one of the main butyrate producing GIT commensals is often decreased, more frequently in CD compared to controls [24, 27]. Our study replicated this finding with reduction in IBD overall, but more so in CD compared to HC, and in active and paediatric CD. Another butyrate producing bacterial genus belonging to the phylum *Firmicutes*, *Eubacterium*, was reduced in IBD in this study. In the current literature, there is slight predominance in UC, but this was not observed in our study, possibly due to fewer studies focusing on UC compared to CD [24]. Additionally, within the *Firmicutes* phylum, both the *Roseburia* genus and the *Lachnospiraceae* family to which it belongs were decreased in IBD/CD/UC compared to HC. This was most striking in *Roseburia*, another butyrate producer [27, 28].

The *Ruminococcus* genus within the *Ruminococcaceae* family of the *Firmicutes* phylum, were all depleted significantly in IBD compared to HC, with *Ruminococcus* being more depleted in CD. At the species level, this mainly pertained to *R. bromii*. On the other hand, *R. gnavus*, a mucin degrading bacteria, was found to be enriched in IBD compared to HC. As a producer of glucorhamnan polysaccharide, which triggers inflammatory pathways through toll-like receptor 4, it has been strongly associated with CD [27, 29, 30].

***Bacteroidetes*** phylum was depleted in IBD whereas its genus *Bacteroides* was depleted in CD but enriched in UC compared to HC. Within the literature, this decrease in IBD was consistent for *Bacteroidetes*, however, increase of *Bacteroides* in UC was not previously noted [25]. More significant *Bacteroides* depletion has been previously associated with more active disease, potentially supporting the idea of greater dysbiosis seen in CD [31]. *Prevotella*, another genus of the *Bacteroidetes* phylum, was also depleted in IBD and particularly CD, without change in UC compared to HC.

In this study, the phylum ***Proteobacteria*** was significantly increased in IBD overall but particularly in CD compared to HC. Furthermore, *Proteobacteria* was increased more in active IBD compared to during remission, and is often associated with more severe CD [27]. Interestingly, the phylum was depleted in oral IBD patient samples compared to their HCs. These findings are well established in existing literature [27, 32]. *Enterobacteriaceae* (family level of the *Proteobacteria* phylum) replicated the changes of being increased in IBD and particularly CD compared to HC. Within this family of particular significance at the genus/species level is *Escherichia-Shigella* and *E. coli*. These were all increased in our study in IBD, but particularly CD compared to HC, with *E. coli* being more abundant in active CD. Although elevated, the *Enterobacteriaceae* family is often non-specific to IBD, instead being a more abundant upper GIT commensal with lower GIT elevation in cases of shorter intestinal transit such as diarrhoea [28]. However, specific pathogenic strains including the adherent-invasive strain of *E. coli* may be truly pathogenic in IBD whereby the microbe is able to adhere to, and invade into the intestinal epithelium producing inflammation [27]. Unfortunately, very few studies assessed bacteria at the strain level, and overall quantification of this was unable to be assessed.

### Oral-gastrointestinal microbiome axis

Typically, the oral and and intestinal microbiome is kept separate by barriers such as stomach and bile acids. However, in both healthy and diseased states, a relationship exists whereby microbiota may translocate between the regions. In healthy states this can regulate immunity but in disease states, pathogenic bacteria can increase [33]. *Fusobacterium* (in particular *Fusobacterium nucleatum*) is one such oral commensal which our study found pathogenically increased in IBD faecal/mucosal samples but decreased in one paediatric, treatment-naïve patient study. *Fusobacterium nucleatum* uses its byproduct, hydrogen sulfide, which prevents colonic utilisation of butyrate, to induce intestinal inflammation [34]. The cause for *Proteobacteria* depletion in oral IBD samples, whilst there is an increase in faecal/mucosal samples is unclear, however, it may serve as an important and easily obtained diagnostic marker. The overgrowth of *Veillonella* in both oral and faecal/mucosal samples in IBD reflects the mechanism of oral overgrowth translating to increased translocation to the gut microbiome. Its lipopolysaccharide can stimulate TNF-α and IL-6, pro-inflammatory cytokines [33].

### Faecal microbiota transplantation

Although the studies examined did not assess patients who had undergone FMT, FMT appears to be an emerging management option for IBD. FMT revegetates the dysbiotic recipient gut microbiome with a diverse microbiome from a healthy donor. A recent Cochrane systematic review found FMT to induce clinical and endoscopic remission in active UC, however, with regards to inducing remission in CD or maintaining remission in UC or CD, the evidence was unclear [11]. Some studies also reported worsening of IBD after FMT and whilst it is safe in the short term, its long-term safety remains unknown [12]. This is supported in the Australian literature and guidelines [35, 36].

However, our study found that there was generally more dysbiosis in CD compared to UC, with an assumption that FMT could be more effective in CD. Due to equivocal findings, it lends to the possibility that there could be other mechanisms which may explain the pathogenesis of IBD, and that the mechanism of CD may possibly differ from that of UC. One explanation is that dysbiosis, through weakening of epithelial tight junctions, may only be the trigger for an immune inflammatory cascade or alternatively be a consequence of IBD. Thus, resolving dysbiosis using FMT may not be effective [37].

### Limitations

Although this study benefited from the use of specific exclusion criteria to restrict confounders and was able to assess various subgroups, it encountered many of the limitations faced by prior systematic reviews within this area.

The studies were laboratory based, and variations in both patient population and methodology existed, as well as variations in bacterial taxonomic results and inconsistent reporting. Whilst next-generation sequencing (NGS) with the use of 16 S rRNA gene sequencing was the analytic technique used across the majority of studies, different regions of the bacterial genome were sequenced which can introduce bias. This was also the case with quantitative polymerase chain reaction (qPCR), the most common non-NGS tool used. Furthermore, despite most studies using faecal samples, other studies using intestinal mucosal biopsies (often taken from variable regions with or without active inflammation) and oral samples (saliva or swabs) were included. This may have contributed to variability of the results.

Comparison of the studies also reflected a major variance in preferential reporting of bacteria. As this was not a meta-analysis, study metadata was not accessed for objective comparison of all bacteria analysed by the authors, not allowing for assessment of bacteria without significant changes that were not discussed by the authors.

Despite excluding studies with 10 or less IBD patients, the majority of studies in this study included low cohort numbers, with less than 30 IBD patients from each study being common. Most studies did not include power calculations, however more patients in the study cohorts would be beneficial given known inter-individual variances in gut microbiome, even in healthy individuals.

Furthermore, the majority (*n* = 57/83) of studies included were cross-sectional, thus isolated to a specific point in time. The remaining studies were longitudinal with *n* = 15/83 being cohort studies and *n* = 11/83 being case-control studies. Given the relatively lower number of longitudinal studies, it is difficult to characterise changes in IBD over time, particularly before and after onset of IBD to deduce whether dysbiosis is a consequence of, or contributor to the pathogenesis of IBD.

Overall, the use of subgroups allowed for separation of these various confounders to ascertain if major changes in gut microbiota was as a result of specific changes in a specific subgroup.

### Future directions

The mechanism of IBD is complex, and its pathogenesis is thought to involve an interplay between dysbiosis with genetic and autoimmune processes. Alongside microbiome studies, the study of genetics as well as immunopathology at both a gut mucosal and circulating level would be needed to better understand the pathogenesis of IBD.

## Conclusion

This study confirmed reduced alpha diversity in IBD and depletion of common protective butyrate producing *Firmicutes* bacteria including *Roseburia* and *Faecalibacterium*. Such changes were more evident in CD, particularly active and paediatric CD. *Ruminococcus* was also reduced in IBD, however its species *R. gnavus*, a pro-inflammatory microbe was enriched, particularly in CD. Whilst an increase of *Bacteroides* in UC has been found, despite reduction in CD, this has not been previously reported and supports reduced dysbiosis in UC compared to CD. *Proteobacteria* and its family *Enterobacteriaceae* are increased in IBD.

## Supplementary Information

Below is the link to the electronic supplementary material.

Supplementary Material 1 (DOCX 311 KB)

## Data Availability

Data is provided within the manuscript or supplementary information files.

## Abbreviations

IBD: Inflammatory bowel disease
CD: Crohn’s disease
UC: Ulcerative colitis
FMT: Faecal microbiota transplantation
HC: Healthy controls
IBD-U: IBD unclassified
GIT: Gastrointestinal tract
SCFA: Short–chain fatty acid
ETBF: Enterotoxic Bacteroides fragilis
PRISMA: Preferred Reporting Items for systematic Reviews and Meta–Analyse
NGS: Next generation DNA sequencing
FISH: Fluorescent in situ hybridization
NOS: Newcastle–Ottawa Scale (NOS)
qPCR: Quantitative polymerase chain reaction
