# Supplementary material for: Is the microbiome the answer to inflammatory bowel disease: systematic review
Source: Langenbecks Arch Surg. 2025 Nov 4;411(1):2. doi: 10.1007/s00423-025-03897-0 (PMC12586227; doi:10.1007/s00423-025-03897-0)
Supplement: Supplementary file 1 — Supplementary Material 1 (DOCX 311 KB) [file 423_2025_3897_MOESM1_ESM.docx]

**Title**: Is the Microbiome the Answer to Inflammatory Bowel Disease: Systematic Review

**Journal Name**: Langenbeck's Archives of Surgery

**Authors**: Devansh Shah, Fiona Phan, Zirong Yu, Joseph Do Woong Choi, James Wei Tatt Toh

**Corresponding Author**: James Wei Tatt Toh

- Email: [james.toh@health.nsw.gov.au](mailto:james.toh@health.nsw.gov.au)
- Affiliations:
  - Department of Colorectal Surgery, Westmead Hospital. Corner Hawkesbury Road and Darcy Roads, Westmead, New South Wales, Australia.
  - Faculty of Medicine and Health, The University of Sydney, Sydney, NSW, Australia.

**Supplementary Text**

***Table S1***: Summary of included study findings. FISH – fluorescence in situ hybridization; qPCR – quantitative PCR; PCA – principal component analysis; PCoA – principal co-ordinate analysis; NMDS – non-metric multidimensional scaling; IBD – inflammatory bowel disease; CD – Crohn’s disease; UC – ulcerative colitis; HC – healthy control; HS – healthy sibling; MSG – metagenomic shotgun sequencing; 16S rRNA GS – 16S rRNA gene sequencing; m-ddPCR - multiplexed droplet digital PCR

| **Author** | **Year** | **Study Design** | **Participants** | | | | | **Age Group** | **Disease Activity** | **Treatment** | **Specimen** | **Analysis Method** | **Findings** | | |
| --- | --- | --- | --- | --- | --- | --- | --- | --- | --- | --- | --- | --- | --- | --- | --- |
|  |  |  | **CD** | **UC** | **UIBD** | **IBD Total** | **HC** |  |  |  |  |  | **Alpha/Beta Diversity** | **Increased Abundance at Taxonomic Level in IBD** | **Decreased Abundance at Taxonomic Level in IBD** |
| Budzinskia et al[38] | 2025 | Cross-sectional study | 14 | 0 | 0 | 14 | 8 | Adult | Not specified | Not specified | Faeces | 16S rRNA gene sequencing | Beta diversity (by Bray-Curtis) significantly different between CD vs HC | Genus level: Escherichia-Shigella Specieis level: Ruminococcus gnavus | Genus level: Faecalibaterium, Lachnospiraceae, Roseburia |
| Zheng et al[39] | 2024 | Cross-sectional study | 174 | 205 | 0 | 379 | 118 | Adult | Not specified | 5-ASA/Mesalazine, steroids, immunomodulator, biologics | Faeces | Multiplexed droplet digital PCR | Alpha diversity (by richness) significantly reduced in CD/UC vs HC. No difference in CD vs UC Beta diversity (by PCoA) significantly different between UC > CD vs HC | **Increased in** **IBD vs HC** Phylum level:  Proteobacteria **Increased in CD vs HC** Species level: Escherichia coli, some Streptococcus species **Increased in UC vs HC** Species level: Bacteroides fragilis, Veillonella parvula, Clostridium spiroforme, Blautia hansenii | **Dereased in** **IBD vs HC** Phylum level:  Firmicutes **Decreased in CD vs UC/HC** Phylum level: Bacteroidetes **Decreased in CD vs HC** Species level: Eubacterium hallii, Blautia obeum, Roseburia inulinivorans, Eubacterium rectale **Decreased in UC vs HC** Species level: Collinsella aerofaciens, Clostridium leptum, Ruminococcus torques, Asaccharobacter celatus, Gemmiger formicilis, Fusicatenibacter saccharivorans |
| Chen et al[40] | 2024 | Cross-sectional study | 52 | 0 | 0 | 52 | 52 | Adult | 52% active | 5-ASA, steroids, immunomodulator, biologics No antibiotics or PPI in last month | Faeces | 16S rRNA gene sequencing | Alpha diversity (by Shannon index) significantly different between CD vs HC. No difference in CD active and remission Beta diversity (by PCoA) significantly different between CD vs HC. No difference in CD active and remission | **Increased in CD vs HC** Phylum level: Proteobacteria | **Decreased in CD vs HC** Phylum level: Firmicutes Genus level: Dorea, Fusicatenibacter |
| Conrad et al[41] | 2024 | Cross-sectional study | 77 | 6 | 29 | 112 | 83 | Paediatric | 46% active | 5-ASA, steroids, immunomodulator, biologics, EEN. Some on PPI, probiotics, antibiotics | Faeces | Metagenomic shotgun sequencing | Alpha diversity (by richness and Shannon index) significantly reduced in IBD vs HC. Beta diversity (by PCoA) significantly different between IBD vs HC | **Increased in IBD vs HC** Species level: Ruminococcus gnavus, Escherichia coli | **Decreased in IBD vs HC** Phylum level: Bacteroidetes Species level: Eubacterium rectale, Alistipes onderdonkii, Alistipes finegoldii |
| Scanu et al[42] | 2024 | Case-control study | 0 | 53 | 0 | 53 | 37 | Adult | Not specified | 77% steroids | Faeces | 16S rRNA gene sequencing | Alpha diversity (by Shannon–Wiener index) no significant difference in UC vs HC. Beta diversity (by Bray-Curtis) significantly different between UC vs HC | **Increased in UC vs HC** Family level:  Enterobacteriaceae, Clostridiaceae Genus level: Bifidobacterium, Streptoccus, Lactobacillus, Veillonella, Enterococcus, Clostridium | **Decreased in UC vs HC** Family level: Ruminococcaceae Genus level: Ruminococcus, Coprococus, Akkermansia |
| Alahdal et al[43] | 2024 | Cohort study | 80 | 0 | 0 | 80 | 15 | Adult | 31% active | No antibiotics or probiotics in last month | Faeces | 16S rDNA gene sequencing | Alpha diversity (by Chao1 and Shannon index) significantly reduced in CD vs HC. Beta diversity (by PCoA) significantly different between CD vs HC | **Increased in CD vs HC** Phylum level: Bacteroidetes, Proteobacteria, Fusobacteria | **Decreased in CD vs HC** Phylum level: Firmicutes, Actinobacteria |
| Han et al[44] | 2024 | Case-control study | 69 | 11 | 0 | 80 | 24 | Adult | Not specified | No antibiotics or probiotics in last 3 months | Faeces, saliva | 16S rRNA gene sequencing | **Faeces and saliva** Alpha diversity (by Chao1) significantly reduced in IBD vs HC Beta diversity (by PCoA) significantly different between IBD vs HC | **Increased in IBD vs HC** *Faeces* Phylum level: Proteobacteria Genus level: Shigella  *Saliva* Phylum level: Proteobacteria, Firmicutes  Genus level: Neisseria | **Decreased in IBD vs HC** *Faeces* Phylum level: Bacteroidetes, Fusobacteria, Actinobacteria  Genus level: Prevotella, Faecalibacterium, Roseburia *Saliva* Phylum level: Fusobacteria, Actinobacteria Genus level: Fusobacterium and Actinomyces |
| Alsulaiman et al[45] | 2023 | Cross-sectional study | 135 | 84 | 0 | 219 | 124 | Adult | Not specified | No antibiotics in last 3 months | Faeces | 16S rRNA gene sequencing | Alpha diversity (by Shannon index) significantly reduced in IBD vs HC. Beta diversity (by PCoA) no significant difference in IBD vs HC (when adjusted for age and sex | **Increased in IBD vs HC** Species level: Blautia hanseni, Clostridium perfringens **Increased in CD vs HC** Genus level: Veillonella, Lachnoclostridium Species level: Blautia hansenii, Clostridium perfringens, Dialister propionicifaciens, Clostridioides difficile **Increased in UC vs HC** Genus level: Lachnoclostridium Species level: Blautia hansenii, Clostridium perfringens | **Decreased in IBD vs HC** Genus level: Prevotella, Bacteroides  **Decreased in CD vs HC** Genus level: Prevotella, Lactobacillus, Bacteroides **Decreased in UC vs HC** Genus level: Prevotella, Bacteroides |
| Markelova et al[46] | 2023 | Cross-sectional study | 96 | 0 | 0 | 96 | 24 | Adult | 68.8% active | 7.29% on no treatment, others on 5-ASA, corticosteroids, immunosuppressor, biologics. | Faeces | 16S rRNA gene sequencing | Alpha diversity (by Shannon index) significantly reduced in CD vs HC Beta diversity (by PCoA) significantly different between CD vs HC | **Increased in CD vs HC** Family level:  Enterobacteriaceae (phylum Proteobacteria) Lactobacillaceae, Enterococcaceae, Streptococcaceae (phylum Firmicutes)  Verrucomicrobiaceae (phylum Verrucomicrobiota) | **Decreased in CD vs HC** Family level:  Rikenallaceae (phylum Bacteroidetes), Clostridiaceae (phylum Firmicutes), Coriobacteriaceae (phylum Actinobacteria) |
| Lopez et al[47] | 2023 | Cross-sectional study | N/S | N/S | 22 | 22 | 5 | Paediatric | Unknown | Steroids, 5-ASA, tacrolimus, methotrexate, azathioprine, anti-TNFa inhibitor (overall patients on or off treatment not specified) 14% of IBD group had antibiotics | Faeces | 16S rRNA gene sequencing | Alpha diversity (by Shannon, Chao1 index) significantly reduced IBD vs HC Beta diversity (by PCA) significantly different between IBD vs HC | Genus level:  Bacteriodes | No differences to report |
| Elmaghrawy et al[48] | 2023 | Cohort study | 94 | 52 | 10 | 156 | 102 | Paediatric | 93% active | All treatment-naïve. No antibiotics in last 2 months. | Tongue/buccal polyurethane sponge swab | 16S rRNA gene sequencing | Alpha diversity (by Chao1, Shannon, Simpson index) not significantly different in IBD vs HC Beta diversity (by PCA) significantly different between IBD vs HC | Genus level:  Ottowia, Pseudopropionobacterium, Lautropia, Staphylococcus, Pseudomonas, Corynebacterium, Eikenella, Streptococcus | Genus level:  Veillonella, Oribacterium, Lachnoanaerobaculum, Prevotella, Fusobacterium, Leptotrichia, Porphyromonas |
| Wu et al[49] | 2023 | Cross-sectional study | 12 | 0 | 0 | 12 | 11 | Paediatric | 58% active | 7/12 (58%) treatment-naïve | Faeces, mucosal biopsies (ileocaecal valve, terminal ileum, transverse colon) | 16S rRNA gene sequencing | Alpha diversity (by Chao1, Observed species, phylogenetic diversity, Shannon index) not significantly different in CD vs HC Beta diversity (by PCoA) not significantly different between CD vs HC | **Increased in active CD vs HC/CD remission Faeces** Genus level: Clostridium sensu stricto, Enterobacter, Akkermansia, Fusobacterium, Streptoccoccus **Mucosa** Ileocaecal valve **Increased in active CD vs HC/CD remission** Genus level: Escherichia-Shigella, Intestinibacter, Lachnoclostridium Terminal ileum **Increased in CD vs HC** Genus level: Escherichia-Shigella  Transverse colon **Increased in active CD vs HC/CD remission** Phylum level: Proteobacteria Genus level: Escherichia-Shigella, Enterococcus | **Decreased in active CD vs HC/CD remission Faeces** Genus level: Holdemanella, Faecalibacterium **Mucosa** Ileocaecal valve + Terminal ileum **Decreased in CD vs HC** Genus level: Ruminococcus, Streptococcus, Fusobacterium, Faecalibacterium  Transverse colon **Decreased in active CD vs HC/CD remission** Phylum level: Firmicutes Genus level:  Bacillus, Blautia, Ruminococcus |
| Al-Amrah et al[50] | 2023 | Cross-sectional study | 6 | 5 | 0 | 11 | 10 | Adult | 72% active | Aminosalicylates, azathioprine, corticosteroids (overall patients on or off treatment not specified) No antibiotics or biological therapy use in last 2 months | Faeces | 16S rRNA gene sequencing & qPCR | Alpha diversity (by Shannon index) significantly reduced in IBD vs HC Beta diversity (by PCA, PCoA) significantly different between CD vs HC | Phylum level:  Proteobacteria, Verrucomicrobia, Fusobacteria Family level:  Bacteroidaceae, Porphyromonadaceae, Verrucomicrobiaceae, Enterobacteriaceae, Streptococcoaceae, Rikenellaceae Lactobacillaceae, Alcaligenaceae, Fusobacteraceae, Veillonellaceae Genus level:  Escherichia, Bacteroides, Sutterella, Proteus, Streptococcus Species level:  Bacteroides fragilis, Escherichia coli, Lactobacillus salivarius | Phylum level:  Firmicutes, Bacteroidetes, Actinobacteria Family level:  Actinomycetaceae, Bifidobacteriaceae, Paraprevotellaceae, Prevotellaceae, Muribaculoceae, Erysipelotrichaceae, Lachnospiraceae, Ruminococcaceae, and Succinivibrionaceae Genus level:  Bifidobacterium, Faecalibacterium, Prevotella, Eubacterium, Coprococcus, Dialister, Roseburia, Ruminococcus, Blautia Species level: Faecalibacterium prausnitzii, Bacteroides plebeius, Bacteroides uniformis, Bifidobacterium adolescentis, Rumincoccus gnavus, Coprococcus eutactus, Prevotella copri, Ruminococcus bromii |
| Räisänen et al[51] | 2023 | Case-control study | N/S | N/S | 21 | 21 | 79 | Paediatric | Unknown | IBD medications not specified. 1/21 had antibiotics in last 3 months. | Saliva | 16S rRNA gene sequencing | Alpha diversity (by Chao1 index) reduced in IBD vs HC Beta diversity (by PCoA) reduced between IBD vs HC | Phylum level: Bacteroidetes, Proteobacteria | Phylum level: Fusobacteria |
| Gao et al[52] | 2023 | Cross-sectional study | 870 | 0 | 0 | 870 | 548 | Adult | 100% active | IBD medications not specified.  No antibiotics in last 3 months. | Faeces | 16S rRNA gene sequencing | Alpha diversity (by Shannon, Simpson, Chao1 index) significantly reduced in CD vs HC  - but not by ACE index Beta diversity (by PCoA) significantly different between CD and HC | **Increased in CD vs HC** Species level:  Escherichia coli, Flavonifractor plautii, Klebsiella pneumoniae | **Decreased in CD vs HC** Species level:  Bacteroides intestinalis, Roseburia intestinalis, Prevotella ruminicola |
| Salimi et al[53] | 2022 | Cross-sectional study | 7 | 8 | 0 | 15 | 15 | Adult | 33% active | IBD medications not specified.  No antibiotics in last 4 weeks. | Faeces | 16S rRNA gene sequencing | Number and diversity of microbiota significantly reduced in IBD vs HC. Alpha/Beta diversity not described. | Phylum level:  y-Proteobacteria Family level:  Enterobacteriaceae Species level: Enterococcus faecalis | Phylum level:  Firmicutes, Bacteroidetes, Actinobacteria Genus level:  Bifidobacterium, Roseburia, Lactobacillus Species level: Faecalibacterium prausnitzii, Lactobacillus species |
| Zhu et al[54] | 2022 | Cross-sectional study | 0 | 40 | 0 | 40 | 20 | Adult | 50% active | IBD medications not specified.  No antibiotics or corticosteroids in last 3 months. | Faeces | 16S rRNA gene sequencing | Alpha diversity (by Chao1, Shannon, observed species) significantly reduced in remission UC vs HC  - no difference based on phylogenetic diversity and no significant difference in active UC vs HC Beta diversity (by PCoA) significantly different between UC vs HC | **Increased in overall UC vs HC** Phylum level:  Patescibacteria Genus level:  Klebsiella, Rothia, Haemophilus **Increased in active UC vs HC** Phylum level:  Proteobacteria Genus level:  Streptococcus, Veillonella **Increased in remission UC vs HC** Genus level:  Blautia, Faecalitalea, Flavonifractor | **Decreased in overall UC vs HC** Phylum level:  Desulfobacterota, Verrucomicrobiota  Genus level:  Butyricicoccus, Lachnoclostridium, Ruminococcus, Lachnospira **Decreased in active UC vs HC** Genus level:  Dorea, Fusicatenibacter, Lactobacillus, Parabacteroides, Roseburia **Decreased in remission UC vs HC** Phylum level:  Synergistota Genus level:  Agathobacter, Alistipes, Coprococcus, Enterococcus |
| Xu et al[55] | 2022 | Cross-sectional study | 0 | 15 | 0 | 15 | 15 | Adult | 0% active All in remission | Not specified | Faeces | 16S rRNA gene sequencing | Alpha diversity (by Chao1, ACE, goods converage, observed species) significantly reduced in UC vs HC Beta diversity (by PCA) significantly reduced in UC vs HC | **Increased in UC vs HC** Phylum level:  Proteobacteria, Actinobacteria, Verrucomicrobia Genus level: Escherichia-Shigella, Bifidobacterium Species level:  Escherichia coli, Klebsiella pneumoniae, Bifidobacterium longum subsp. Longum, Bacteroides ovatus V975 | **Decreased in UC vs HC**  Phylum level: Firmicutes, Fusobacteria, Bacteroidetes Genus level: Bacteroides, Dialister, Subdoligradulum, Ruminococcus Species level: Uncultured Bacteroides sp. |
| Hu et al[56] | 2022 | Cross-sectional study | 91 | 0 | 0 | 91 | 91 | Adult | 100% active | Aminosalicyclic acid, cortocosteroids, immunomodulators, biologics (overall patients on or off treatment not specified). No antibiotics in last 1 month. | Faeces | 16S rRNA gene sequencing | Alpha diversity (by Shannon index, observed species, phylogenetic diversity) significantly reduced in remission CD vs HC Beta diversity (by PCoA) significantly different between CD vs HC | **Increased in CD vs HC** Phylum level:  Proteobacteria Family level: Enterobacteriaceae Fusobacteriaceae Genus level:  Escherichia-Shigella, Atlantibacter Fusobacterium Veillonella | **Decreased in CD vs HC** Phylum level:  Firmicutes, Bacteroidetes Family level: Lachnospiraceae, Ruminococcaceae Prevotellaceae Genus level:  Roseburia, Agathobacter, Lachnospira Faecalibacterium Romboutsia Prevotella |
| Ma et al[30] | 2022 | Cross-sectional study | 40 | 0 | 0 | 40 | 30 | Adult | Not specified | No IBD medications for past 2 months, including antibiotics or probiotics | Faeces | 16S rRNA gene sequencing | Alpha diversity (by observed species) significantly reduced CD vs HC  - also significantly reduced (by Shannon, Simpson, Chao1) in advanced CD vs HC (but not early CD vs HC) Beta diversity (by PCoA) significantly different in CD vs HC | **Increased in CD vs HC**  Phylum level:  Bacteroidetes Genus level: Escherichia-Shigella, Proteus | **Decreased in CD vs HC**  Phylum level:  Firmicutes |
| Jacobs et al[57] | 2022 | Cross-sectional study | 88 | 0 | 0 | 88 | 110 | Adult | 0% active All in remission | 5-aminosalicylate, immunomodulator, biologics (overall patients on or off treatment not specified). | Faeces | 16S rRNA gene sequencing | Alpha diversity (by Shannon, Chao1 index) significantly reduced in CD vs HC Beta diversity (by PCoA) significantly different between CD vs HC | **Increased in CD vs HC** Genus level:  Bacteroides, Escherichia-Shigella, Fusobacterium, Staphylococcus, Streptococcus, Rothia, Mycoplasma | **Decreased in CD vs HC** Genus level: Akkermansia, Prevotella, Faecalibacterium |
| Sternes et al[58] | 2022 | Cross-sectional study | 0 | 0 | 64 | 64 | 105 | Adult | Not specified | Disease modifying anti-rheumatic drugs, anti-TNFa inhibitor, corticosteroids (overall patients on or off treatment not specified) No antibiotics or probiotics in last 6 months. | Faeces, mucosal biopsies (terminal ileum, right colon, rectum) | 16S rRNA gene sequencing | Alpha diversity (by observed species) significantly reduced in IBD vs HC Beta diversity (by PCoA) significantly different between IBD vs HC | Genus level: Streptococcus, Haemophilus, Ruminococcus, Fusobacterium, Shigella | Order level: Clostridales Genus level: Faecalibacterium, Roseburia |
| Paljetak et al[59] | 2022 | Cross-sectional study | 10 | 13 | 0 | 23 | 12 | Adult | 95% active | Treatment-naïve No antibiotics in last 3 months | Faeces, mucosal biopsies (terminal ileum to rectum)   - mucosal samples not comparing IBD vs HC so excluded | 16S rRNA gene sequencing | Not assessed for IBD vs HC | **Increased in IBD vs HC** Genus level:  Bacteroides  **Increased in UC vs HC** Family level: Veillonellaceae, Fusobacteriaceae Genus level: Veillonella, Fusobacterium | **Decreased in IBD vs HC** Family level: Ruminococcaceae, Verrucomicrobiaceae Genus level:  Anaerostipes, Ruminococcus  **Decreased in CD vs HC** Family level: Veillonellaceae |
| Hu et al[60] | 2022 | Cross-sectional study | 41 | 0 | 0 | 41 | 24 | Adult | Not specified | Corticosteroids, mesalazine, azathioprine, biologics, tacrolimus, sulphasalazine (overall patients on or off treatment not specified). No antibiotics or probiotics in last 3 months. | Saliva | 16S rRNA gene sequencing | Alpha diversity (by Shannon index) not significantly different in CD vs HC Beta diversity (by PCoA) significantly different between CD vs HC | **Increased in CD vs HC** Phylum level:  Actinobacteria, Proteobacteria Species level: Ruminococcus gnavus, Blautia species, Clostridium innocuum | **Decreased in CD vs HC** Species level: Bacteroides vulgatus, Bacteroides plebeius, Bacteroides stercoris, Rominococcus torques, Roseburia inulinivorans, Faecalibacterium prausnitzii |
| Zuo et al[61] | 2022 | Cross-sectional study | 0 | 19 | 0 | 19 | 23 | Paediatric | 100% active | Corticosteroids, biologics, immunomodulator, 5-ASA (overall patients on or off treatment not specified). No antibiotic or probiotic use during study | Faeces | 16S rRNA gene sequencing& metagenomic shotgun sequencing | Alpha diversity (by Shannon index) significantly reduced in UC vs HC Beta diversity (by PCoA) significantly different between UC vs HC | **Inceased in UC vs HC** Family level:  Streptococcaceae, Enterobacteriaceae, Bacteroidaceae, Bifidobacteriaceae | **Decerased in UC vs HC** Family level:  Ruminococcaceae, Akkermansiaceae, Clostridiaceae, Eggerthellaceae, Lachnospiraceae, Oscillospiraceae |
| Park et al[62] | 2022 | Cohort study | 10 | 9 | 0 | 19 | 19 | Adult | Not specified | 5-ASA, immunomodulators, steroids (overall patients on or off treatment not specified). Anti-TNFa therapy started after 1st visit. No antibiotics or probiotics in last 3 months. | Faeces, saliva, serum, urine | 16S rRNA gene sequencing (faeces & saliva), Extracellular vesicles (faeces, saliva, serum, urine) | Alpha diversity (by ACE, Chao1, Jackknife, Shannon, Simpson index) of faeces - significantly reduced in IBD vs HC Beta diversity (by PCoA) of faeces - no significant difference in IBD vs HC | **Increased in IBD vs HC** (before anti-TNFa therapy) In faeces/saliva by 16S rRNA sequencing Family level: Enterococcaceae  Species level: Enterococcus faecium After anti-TNFa therapy - levels more similar to HC In faeces by extracellular vesicles Genus level: Veillonella, Enterococcus Species level: Clostridiodes difficile | **Decreased in IBD vs HC** (before anti-TNFa therapy) In faeces/saliva by 16S rRNA sequencing Phylum level: Actinobacteria Genus level: Ruminococcus  After anti-TNFa therapy these levels more similar to HC In faeces by extracellular vesicles Phylum level: Proteobacteria  Genus level: Ruminococcus Species level: Acidovorax caeni, Enterococcus faecalis |
| Berbisá et al[22] | 2022 | Cross-sectional study | 0 | 41 | 0 | 41 | 144 | Adult | Active 42% | 3/41 (7%) on no treatment. Remaining on 5 ASA, immunosuppressants, biologicals, combination therapy. No antibiotics in last 3 months. | Faeces | 16S rRNA gene sequencing | Alpha diversity (by Shannon index) not significantly different in UC vs HC Beta diversity (by PCoA) not significantly different in UC vs HC | **Increased in UC vs HC** Genus level: Ruminiclostridium | **Decreased in UC vs HC** Phylum level: Verrucomicrobia Family level: Veillonellaceae Genus level: Akkarmansia Coprococcus, Lachnospiraceae, Dialister |
| Barberio et al[63] | 2022 | Cross-sectional study | 0 | 46 | 0 | 46 | 36 | Adult | 43% active | Mesalizine (overall patients on or off treatment not specified). No antibiotics or probiotics during study. | Faeces | 16S rRNA gene sequencing | Alpha diversity (by Shannon index) significantly reduced in active UC > remission UC vs HC Beta diversity (by PCoA) significantly different between active UC vs remission UC vs HC | **Increased in overall UC vs HC** -  Phylum level: Actinobacteria Genus level: Blautia, Dorea **Increased in active UC vs HC** Phylum level: Proteobacteria  Species level: Haemophilus parainfluenzae, Streptococcus anginosus, Clostridium symbiosum **Increased in remission UC vs HC** Species level: Bifidobacterium adolescentis, Ruminococcus gnavus | **Decreased in UC vs HC** Phylum level: Tenericutes, Verrucomicrobia, Euryarchaeota,Cyanobacteria **Decreased in active UC vs HC** Genus level: Lachnospira, Oscillospira |
| Sukhina et al[64] | 2022 | Cross-sectional study | 0 | 0 | 157 | 157 | 150 | Adult | Not specified | Not specified | Faeces | 16S rRNA gene sequencing, metagenomic shotgun sequencing | Not assessed | **Increased in IBD vs HC** Phylum level:  Proteobacteriae Family level:  Sphingomonadoceae, Burkholderiaceae, Xanthomonadoceae, Comamonadoceae | **Decreased in IBD vs HC** Phylum level:  Firmicutes Family level:  Enterobacteriaceae, Ruminococcaceae, Lachnospiraceae |
| Teofani et al[65] | 2022 | Cross-sectional study | 52 | 58 | 0 | 110 | 42 | Adult | 32% active | IBD medications not specified. No antibiotics in last 4 weeks. | Faeces | 16S rRNA gene sequencing analysis V3-4 regions | Alpha diversity (by Shannon, Chao1, Simpson index) significantly reduced in IBD vs HC Beta diversity (by PCoA) significantly different between IBD vs HC | **Increased in IBD vs HC** Family level:  Coriobacteriaceae, Streptococcaceae **Increased in UC vs HC** Family level:  Atopobiaceae, Bifidobacteriaceae, Defluvitillaceae | **Decreased in IBD vs HC** Family level:  Christensenellaceae, Desulfovibrionellaceae, Marinifilaceae, Rikenellaceae, Ruminococcaceae, Tannerelleaceae, Barneselliaceae. **Decreased in UC vs HC** Family level:  Atopobiaceae, Bifidobacteriaceae, Defluvitillaceae |
| Wan et al[66] | 2022 | Cross-sectional study | 0 | 29 | 0 | 29 | 30 | Adult | Not specified | IBD medications not specified.  No antibiotics in last 3 months. | Faeces | 16S rRNA gene sequencing | Alpha diversity (by observed OTUs, Shannon, Chao1, Simpson index) significantly reduced in UC vs HC Beta diversity (by PCoA) no significant difference between UC vs HC | **Increased in UC vs HC** Phylum level:  Proteobacteriae Genus level:  Escherichia-Shigella (phylum Proteobacteria) | **Decreased in UC vs HC** Phylum level:  Actinobacteria Genus level:  Bifidobacterium, Roseburia, Ruminococcus, Clostridiales |
| Wang et al[67] | 2022 | Case-control study | 0 | 0 | 18 | 18 | 30 | Adult | Not specified | No immune inhibitors or biological medications in last 3 months; other IBD medications not specified.  No antibiotics in last 3 months. | Faeces | 16S rRNA gene sequencing | Alpha diversity (by evennes index) significantly reduced in IBD vs HC  - but not Shannon index Beta diversity (by PCoA) significant difference between IBD vs HC | Phylum level: Proteobacteria, Fusobacteria Genus level: Escherichia-Shigella, Fusobacterium | Phylum level: Firmicutes Species level: Eubacterium coprostanoligenes, Eubacterium hallii group, Alistipes, Erysipelotrichaceae_UCG_003, Fusicatenibacter, Pseudobutyrivibrio, Ruminococcaceae_UCG_013, Subdoligranulum |
| Hu et al[68] | 2021 | Case-control study | 25 | 0 | 0 | 25 | 25 | Adult | 40% Active | Corticosteroids, mesalazine, azathioprine, biologics, tacrolimus, sulphasalazine (overall patients on or off treatment not specified) No antibiotics or probiotics in last month | Faeces, saliva | Metagenomic shotgun sequencing | **Faeces** Alpha diversity (by Shannon, Simpson index) not different in CD vs HC Beta diversity (by PCoA) significantly different between CD vs HC **Saliva** Alpha diversity (by Shannon, Simpson index) not different in CD vs HC Beta diversity (by PCoA) not different between CD vs HC | **Increased in CD vs HC**  Faeces Species level:  Clostridium nexile, Ruminococcus gnavus Saliva No significant differences | **Decrease in CD vs HC**  Faeces Species level:  Faecalibacterium prausnitzzi, Roseburia inulinivorans, Alistipes senegalensis Saliva No significant differences |
| Tang et al[69] | 2021 | Case-control study | 0 | 30 | 0 | 30 | 10 | Adult | 100% active | No antibiotics or IBD medications in last 4 weeks | Faeces | 16S rRNA gene sequencing | Alpha diversity (by Shannon, Simpson index) significantly reduced in UC vs HC Beta diversity (by PCA) significantly different between UC vs HC | **Increased in UC vs HC**  Phylum level: Tenericutes Genus level:  Bacteroides, Escherichia, Sutterella | **Decreased in UC vs HC** Phylum level: Firmicutes Genus level:  Veillonella, Ruminococcus, Coprococcus Species level:  Synergistetes |
| Ostrowski et al[70] | 2021 | Cross-sectional study | 24 | 0 | 0 | 24 | 19 | Adult | Not specified | Azathioprine, corticosteroids, biologics (overall patients on or off treatment not specified). No antibiotics in last 6 months. | Mucosal biopsy (gastric), gastric fluid | 16S rRNA gene sequencing | Alpha diversity (by Shannon index) not significantly different in CD vs HC Beta diversity (by PCoA) significantly different between CD vs HC | **Increased in CD vs HC** Phylum level:  Firmicutes, Actinobacteria, Cyanobacteria | **Decreased in CD vs HC** Phylum level:  Bacteroidetes, Proteobacteria, Fusobacteriota, Campilobacterota, Patescibacteria |
| Liu et al[71] | 2021 | Cross-sectional study | 0 | 48 | 0 | 48 | 48 | Adult | 100% active | IBD medications not specified. No antibiotics or probiotics in last 3 months. | Faeces | 16S rRNA gene sequencing | Alpha diversity (by Shannon, Chao1 index) significantly reduced in Uyghur UC vs Uyghur HC  - no significant change in Han UC vs Han HC Beta diversity (by PCoA) significantly different between Ughur UC vs Ughur HC vs Han UC vs Han HC | **Increased in UC vs HC** Uyghur + Han group  Phylum level:  Proteobacteria, Bacteroidetes Genus level:  Faecalibacterium, Bacteroides, Prevotella Han group Phylum level:  Actinobacteria | **Decreased in UC vs HC** Uyghur + Han group  Phylum level:  Firmicutes Genus level:  Agathobacter, Blautia, Klebsiella, Dorea Uyghur group Phylum level:  Actinobacteria |
| Frau et al[72] | 2021 | Cohort study | 49 | 20 | 0 | 69 | 35 | Adult | 66% active | Mesalazine, corticosteoids, immunosuppressants, biologics (overall patients on or off treatment not specified). 4.3% (3/69) with use of antibiotics in last 3 months. | Faeces, mucosal biopsies (terminal ileum, transverse colon, sigmoid colon) | 16S rRNA gene sequencing | Alpha diversity (by Shannon index) significantly reduced in CD vs HC (faeces and mucosa)  - no significant change in UC vs HC Beta diversity (by PCoA) significantly different between CD vs UC vs HC | **Increased in CD vs HC** Genus level: Bacteroides, Lachnoclostridium | **Decreased in CD vs HC** Genus level: Faecalibacterium, Roseburia |
| Liang et al[73] | 2021 | Cross-sectional study | 0 | 0 | 385 | 385 | 116 | Adult | Not specified | Not specified | Faeces | Metagenomic shotgun sequencing | Alpha diversity (by evenness) lower in IBD vs HC. No significant difference when by Shannon index. Beta diversity (by PCoA) significant difference between IBD vs HC | Phylum level:  Firmicutes, Proteobacteria, Verrucomicrobia Genus level:  Faecalibacterium, Eubacterium | Phylum level:  Bacteroidetes  Genus level:  Alistipes, Parabacteroides, Prevotella, Subdoligranulum, Barnesiella, Akkermansia |
| Nishihara et al[74] | 2021 | Cohort study | 0 | 51 | 0 | 51 | 7 | Adult | 52% active | 5-ASA, corticosteroids, azathioprine, 6-mercaptopurine, anti-TNFa inhibitor (overall patients on or off treatment not specified). No antibiotics in last 3 months. | Mucosal biopsies (rectum, colon) | 16S rRNA gene sequencing | Alpha diversity (by Shannon, Chao1, whole-tree phylogenetic diversity index): significantly reduced in UC, particularly relapse UC vs HC Beta diversity (by PCoA): significantly different between UC vs HC | **Increased in active UC vs remission** Family level: Enterobacteriaceae **Increased in relapsed UC vs non-relapse** Genus level:  Bacteroides | **Decreased in active UC vs remission** Genus level: Prevotella **Decreased in relapsed UC vs non-relapse** Order level:  Clostridiales |
| Xia et al[75] | 2021 | Cross-sectional study | 112 | 122 | 0 | 232 | 145 | Adult | Not specified | Not specified | Faeces | Metagenomic shotgun sequencing | Alpha diversity (by Shannon, Simpson index) reduced significantly in IBD, especially CD vs HC Reduced in UC vs HC but only in American sub-cohort Beta diversity (by PCoA) significantly different in CD more than UC compared to HC | **Increased in CD vs HC** Phylum level:  Firmicutes  Genus level:  Blautia, Escherichia | **Decreased in CD vs HC** Phylum level:  Bacteroidetes Family level:  Eubacteriaceae, Ruminococcaceae Genus level:  Eubacterium, Subdoligranulum |
| Maldonado-Arriaga et al[76] | 2021 | Cross-sectional study | 0 | 18 | 0 | 18 | 15 | Adult | 50% active | 7/18 (38.9%) on no treatment. Others on mesalazine, corticosteroids, anti-TNFa inhibitor. No antibiotics in last 4 weeks. | Faeces | 16S rRNA gene sequencing | Alpha diversity (by observed OTUs, Chao, Simpson, Shannon index): significantly reduced richness in active UC vs remission UC/HC  Beta diversity (by PCA): significantly different between active UC vs remission UC/HC | **Increased in active UC vs remission UC/HC** Phylum level:  Proteobacteria, Fusobacteria Genus level:  Bilophilia, Fusobacterium **Increassed in remission UC vs HC** Genus level:  Bacteroides | **Decreased in active UC vs remission UC/HC** Phylum level: Firmicutes, Bacteroidetes Genus level: Lactobacillus, Faecalibacterium, Roseburia **Decreased in remission UC vs HC** Genus level: Faecalibacterium, Roseburia |
| Dai et al[77] | 2021 | Cross-sectional study | 0 | 16 | 0 | 16 | 10 | Adult | Not specified | IBD medications not specified.  No antibiotics in last 3 months. | Faeces | 16S rRNA gene sequencing | Alpha diversity (by Shannon, Chao1 index) significantly reduced in UC vs HC Beta diversity (via PCoA) significantly different between UC vs HC | **Increased in UC vs HC** Phylum level:  Proteobacteria, Actinobacteria, Firmicutes Class/order level:  Bacilli, Gammaproteobacteria, Actinobacteria Family level:  Enterococcaceae, Lactobacillaceae  Genus level:  Enterococcus, Lactobacillus, Escherichia-Shigella, Bifidobacterium | **Decreased in UC vs HC** Phylum level:  Bacteroidetes Class/order level:  Clostridia, Bacteroidia Family level:  Lachnospiraceae, Bacteroidaceae  Genus level:  Bacteroides |
| Chang et al[78] | 2021 | Cross-sectional study | 6 | 14 | 0 | 20 | 48 | Adult | 30% active | 1/20 (5%) on no IBD medications. Others on mesalizine, corticosteroids, biologics. No antibiotics or probiotics in last 4 weeks. | Faeces | 16S rRNA gene sequencing | Alpha diversity (by Shannon, Simpson, Chao index & observed OTUs): significantly reduced richness and evenness in IBD vs HC; diversity significantly reduced in CD vs HC whereas UC vs HC not as significant Beta diversity (by PCoA): significantly different in IBD vs HC; significant differences separately in CD vs HC and UC vs HC | **Increased in IBD vs HC** Phylum level:  Firmicutes Class level:  Bacilli, Actinobacteria  Genus level:  Bifidobacterium, Ruminococcus gnavus, Streptococcus, Blautia | **Decreased in IBD vs HC** Phylum level: Bacteroidetes Class level:  Bacteroidia, Deltaproteobacteria Family level:  Ruminococcaceae Genus level:  Faecalibacterium, Subdoligranulum, Parabacteroides, Paraprevotella |
| Abdul-Hussein et al[79] | 2021 | Cross-sectional study | 0 | 0 | 50 | 50 | 50 | Adult | Not specified | Not specified | Faeces | Culture using selective growth media, gram stain, catalase, oxidase testing | Not assessed | **Increased in IBD vs HC** Species level:  Lactobacillus species, Escherichia coli | **Decreased in IBD vs HC** Species level:  Citrobacter species |
| Juyal et al[80] | 2021 | Cross-sectional study | 0 | 105 | 0 | 105 | 36 | Adult | 52.3% active | IBD medications not specified. No antibiotics in last 1 month. | Faeces | 16S rRNA gene sequencing | Alpha diversity (by Shannon, Chao1 index) significantly reduced in UC vs HC Beta diversity (by PCoA) significantly different between UC vs HC | **Increased in UC vs HC** Genus level:  Streptococcus, Bifidobacterium, Lactobacillus (lactate producing bacteria) **Increased in newly diagnosed UC vs HC** Genus level:  Escherichia-Shigella, Enterococcus, Faecalibacterium | **Decreased in UC vs HC** Family level:  Lachnospiraceae, Ruminococcaceae (butyrate-producing bacteria) |
| Sanchis-Artero et al[81] | 2021 | Cohort study | 27 | 0 | 0 | 27 | 16 | Adult | Not specified | 5-ASA, azathioprine, corticosteroids, methotrexate (overall patients on or off treatment not specified). No antibiotics or probiotics in last 4 weeks. | Faeces | 16S rRNA gene sequencing | Alpha diversity (by Shannon, Chao index) significantly reduced in CD vs HC Beta diversity (by PCoA) significantly different between CD vs HC | Increased in CD vs HC [before anti-TNFa therapy] Phylum level:  Proteobacteria, Actinobacteria, Fusobacteria Family level:  Enterobacteriaceae, Erysipelotrichaceae, Bifidobacteriaceae Genus level:  Blautia, Escherichia-Shigella, Bifidobacterium, Lachnoclostridium | **Decreased in CD vs HC** [before anti-TNFa therapy] Family level:  Ruminococcaceae, Christensenellaceae Genus level:  Ruminococcus, Agathobacter, Dorea, Fusicatenibacter Species level:  Bacteroides plebeius, Alistipes obesi, Gabonia massiliensis, Faecalibacterium prausnitzii |
| Somineni et al[82] | 2021 | Cohort study | 30 | 17 | 0 | 47 | 18 | Paediatric | 45% active | 26/57 (55%) treatment-naïve. No antibiotic therapy. | Faeces, saliva | 16S rRNA gene sequencing | Alpha diversity (by Shannon, Simpson, alpha, Chao index):  Reduced diversity and richness in stool in IBD vs HC Reduced richness in saliva in IBD vs HC Beta diversity (by PCoA) significantly different between IBD vs HC | **Increased in IBD (oral sites) vs HC** Phylum level:  Actinobacteria, Bacteriodetes, Spirochaetes | **Decreased in IBD (oral sites) vs HC** Phylum level:  Fusobacteria, Firmicutes, Proteobacteria |
| Alam et al[83] | 2020 | Cross-sectional study | 9 | 11 | 0 | 20 | 10 | Adult | Not specified | Not specified | Faeces | 16S rRNA gene sequencing | Alpha diversity (by Shannon index) significantly reduced in CD vs UC/HC. Beta diversity not assessed | **Increased in IBD vs HC**  Phylum level: Firmicutes, Actinobacteria Family level: Coriobacteriaceae (phylum Actinobateria) Burkholderiaceae (phylum Proteobacteria) **Increased in CD vs HC** Phylum level:  Proteobacteria Class level:  Negativicutes, Bacilli (phylum Firmicutes) Family level:  Enterobacteriaceae (phylum Proteobacteria) Prevotellaceae (phylum Bacteroidota) Veillonellaceae, lactobacillaceae, Acidaminococcaceae, Streptococcaceae, Peptostreptococcaceae (phylum Firmicutes) **Increased in UC vs HC** Class level:  Clostridia, Negativicutes, Bacilli (phylum Firmicutes) Family level:  Ruminococcaceae, Lachnospiraceae, Veillonellaceae, Streptococcaceae, Peptostreptococcaceae (phylum Firmicutes) | **Decreased in IBD vs HC**  Family level:  Bifidobacteriaceae **Decreased in CD vs HC** Phylum level:  Bacteroidetes Class level: Clostridia, Erysipelotrichia (phylum Firmicutes) Family level: Ruminococcaceae, Christensenellaceae, Erysipelotrichaceae (phylum Firmicutes) **Decreased in UC vs HC**  Phylum level:  Proteobacteria Family level:  Enterobacteriaceae (phylum Proteobacteria) Acidaminococcaceae, Christensenellaceae, Lactobacilliaceae (phylum Firmicutes) |
| Sila et al[84] | 2020 | Cross-sectional study | 13 | 6 | 0 | 19 | 39 | Paediatric | 100% active | All treatment-naïve | Faeces | 16S rRNA gene sequencing | Alpha diversity (by Shannon index) significantly reduced in IBD vs HS/HC  Beta diversity not assessed | **Increased in IBD vs HS/HC** Phylum level:  Proteobacteria Genus level:  Streptococcus, Lactococcus, Enterococcus, Enterobacter, Citrobacter, Escherichia, Klebsiella | **Decreased in IBD vs HS/HC** Phylum level:  Firmicutes  Genus level:  Clostridium, Paenibacillus, Bacillus, Lactobacillus, Blautia, Eubacterium, Roseburia, Ruminoccocus |
| Qiu et al[85] | 2020 | Cross-sectional study | 25 | 0 | 0 | 25 | 20 | Adult | 76% active | 15/25 (60%) on 5-ASA, corticosteroids, Azathioprine 10/25 (40%) on no treatment No antibiotics or probiotics in last 8 weeks | Faeces | 16S rRNA gene sequencing | Alpha diversity (by Shannon, ACE, Chao1 index) significantly reduced in CD vs HC  Beta diversity not assessed | **Increased CD vs HC** Phylum level:  Proteobacteria Genus level:  Escherichia-Shigella | **Decreased CD vs HC** Phylum level:  Firmicutes, Actinobacteria Genus level:  Faecalibacterium, Gemmiger, Bifidobacterium, Romboutsia, Ruminococcus, Roseburia, Fusicatenibacter |
| Kowalska-Duplaga et al[86] | 2019 | Cross-sectional study | 64 | 0 | 0 | 64 | 18 | Paediatric | 100% active | All treatment-naïve. No antibiotics or probiotics in last 3 months. | Faeces | 16S rRNA gene sequencing | Alpha diversity (by Shannon, observed OTUs, Faith’s Phylogenetic Diversity, Pielou’s evenness index) significantly reduced in CD vs HC Beta diversity (by PCoA) significantly different in CD vs HC | **Increased in CD vs HC** Genus level:  Enterococcus | **Decreased in CD vs HC** Genus (species) level: Bifidobacterium (B. adolescentis), Adlercreutzia, Clostridium (C. celatum), Coprococcus, Roseburia (R. faecis), Faecalibacterium (F. prausnitzii), Gemmiger (G. formicilis), Ruminococcus (R. bromii) |
| Guo et al[87] | 2019 | Cross-sectional study | 95 | 81 | 0 | 176 | 105 | Adult | 71.6% active | No treatment including antibiotics for last 3 months | Faeces | QPCR | Not assessed | **Increased CD vs UC/HC** Species level: Fusobacterium nucleatum, Escherichia coli | **Decreased in IBD vs HC** Species level: Faecalibacterium prausnitzii |
| Malham et al[88] | 2019 | Cross-sectional study | 77 | 58 | 8 | 143 | 34 | Paediatric | Not specified | Immunomodulators, 5-ASA, biologic therapy (overall patients on or off treatment not specified).  3/143 (2%) IBD patients had antibiotic use 3 months of study. | Faeces | 16S rRNA gene sequencing | Alpha diversity (by OTU) significantly reduced in IBD vs HC Beta diversity (by PCoA) no significant different between IBD vs HC nor between IBD subtypes | **Increased in IBD vs HC** Species level:  Flavonifractor plautii | **Decreased in IBD vs HC** Species level: Akkermansia muciniphila, Gemmiger formicilis, Ruminococcus bromii, Clostridium cellulovorans, Collinsella aerofaciens, Bacteroides massiliensis, Adlercreutzia equolifaciens **Decreased in UC vs HC** Species level: Alistipes shahii, Odoribacter planchnicus, Turicibacter sanguinis **Decresed in UC vs CD/HC** Species level:  Coprobacter fastidiosus |
| Olbjørn et al[89] | 2019 | Case-control study | 80 | 27 | 3 | 110 | 75 | Paediatric | Not specified | Treatment-naïve No antibiotics in last 6 months | Faeces | 16S rRNA gene sequencing | Not assessed | **Increased in IBD vs HC** Genus level:  Prevotella **Increased in UC vs CD** Species level:  Mycoplasma hominis | **Decreased in IBD vs HC** Phylum level:  Actinobacteria, Firmicutes, Proteobactaria, Verrucomicrobia Genus level:  Bacteroides, Clostridium, Ruminococcus, Streptococcus, Eschericia-Shigella |
| Kansal et al[90] | 2019 | Cohort study | 138 | 0 | 0 | 138 | 66 | Paediatric | 91.3% active | 88/138 (63.8%) treatment-naïve  No antibiotics in last 3 months. | Mucosal biopsies (terminal ileum, colon - inflamed and non-inflamed regions) | 16S rRNA gene sequencing | Alpha diversity (by Shannon, Chao1, qstat index): significantly reduced in CD especially first diagnosis vs HC - no significant differences between CD subgroups (first diagnosis/remission/relapse) Beta diversity (by PCA): significantly different between first diagnosis CD vs HC Bacterial communities significantly different between first diagnosis/relapse/remission CD. | **Increased in first diagnosis CD vs HC** Species level: Fusobacterium species, Haemophilus parainfluenzae, Veillonella species, Clostridium species, Epulopiscium species, Propionibacterium acnes **Increased in relapse/remission CD vs first diagnosis CD/HC**  Species level: Hespellia porcina, Eubacterium fissicatena | No differences to report |
| Zhong et al[91] | 2019 | Cross-sectional study | 0 | 28 | 0 | 28 | 16 | Adult | 57.1% active | 8/28 (29.6%) not using medications during study. No antibiotics or probiotics in last 2 months. | Mucosal biopsy (rectosigmoid) | FISH (fluorescence in situ hybridization) | Not assessed | **Increased in UC vs HC** Genus level: Escherichia coli, Clostridium, Bacteroides | **Decreased in UC vs HC** Genus level: Lactobacillus, Bifidobacteria |
| Al-Bayati et al[92] | 2018 | Cross-sectional study | 0 | 40 | 0 | 40 | 40 | Adult | Not specified | No corticosteroids in last 4 weeks, otherwise IBD medications not specified. No antibiotics in last 3 months. | Mucosal biopsies (colon, rectum) | 16S rRNA gene sequencing | Not assessed | No differences to report | **Decreased in UC vs HC** Species level: Prevotella spp, Faecalibacterium prausnitzii, Peptostreptococcus productus, Clostridium clostridioforme, Eubacterium rectale, Faecalibacterium prausnitzii, Bifidobacteria, Lactobacilli, Clostridium butyricum |
| Xun et al[93] | 2018 | Case-control study | 13 | 54 | 0 | 67 | 25 | Adult | 32.8% active | 6.0% Treatment-naïve. No antibiotics or probiotics in last 4 weeks. | Saliva | 16S rRNA gene sequencing | Alpha diversity: No signficant difference between UC/CD/HC Beta diversity: Significant difference (by PCoA) between UC/CD/HC | **Increased in UC vs HC** Phylum level:  Firmicutes, Bacteroidetes Family level: Streptococcaceae, Burkholderiaceae, Corynebacteriaceae, Cardiobacteriaceae, Enterobacteriaceae Genus level: Streptococcus, Lautropia, Corynebacterium, Cardiobacterium, Acinetobacter **Increased in CD vs HC** Family level:  Veillonellaceae, Coriobacteriaceae, Pseudomonadaceae  Genus level: Veillonella, Selenomonas | **Decreased in IBD vs HC** Family level: Porphyromonadaceae, Planococcaceae,  Genus Level: Porphyromonas, Anaerovorax **Decreased in UC vs HC** Family level: Paraprevotellaceae, Lachnospiraceae **Decreased in CD vs HC** Family level:  Neisseriaceae, Micrococcaceae, Pasteurellaceae, Carnobacteriaceae, Mogibacteriaceae, Peptostreptococcaceae, Tissierellaceae, Mycoplasmataceae |
| de Meij et al[94] | 2018 | Cohort study | 63 | 41 | 0 | 104 | 61 | Paediatric | 100% active | All treatment-naïve. No antibiotics in last 1 month. | Faeces | QPCR | Alpha diversity (by Shannon index): not significantly different between IBD vs HC Beta diversity (by PCoA): different between IBD vs HC | **Increased in IBD vs HC** Species level:  Escherichia coli | **Decreased in IBD vs HC** Phylum level:  Bacteroidetes Species level:  Alistipes finegoldii, Alistipes putredinis, FAFV (Firmicutes, Actinobacteria, Fusobacteria, Verrucomicrobia) species, Prevotella species **Decreased in UC vs CD/HC** Species level:  Bacteroides fragilis, Akkermansia muciniphila |
| Walujkar et al[95] | 2018 | Cohort study | 0 | 12 | 0 | 12 | 7 | Adult | 100% active | No IBD treatment or antibiotics in last 3 months. | Mucosal biopsies (colon - at site of inflammation) | 16S rRNA gene sequencing, quantitative PCR | Alpha diversity (by Chao1, Shannon, Inverse simpson, observed species index): decreased in UC vs HC especially in active stage Beta diversity (by NMDS): significantly different between UC vs HC | **Increased in UC vs HC** Genus level: Achromobacter, Stenotrophomonas, Ochrobactrum, Pseudomonas **Increased in active UC vs remission/HC** Phylum level:  Proteobacteria | **Decreased in UC vs HC** Genus level:  Dialister, Faecalibacterium, Megaspherae, Oscillospira, Roseburia  **Decreased in active UC vs remission/HC** Phylum level:  Firmicutes and Bacteroidetes |
| Ma et al[96] | 2018 | Cross-sectional study | 15 | 14 | 0 | 29 | 13 | Adult | 86.2% active | Steroids, azathioprine, 5-ASA (overall patients on or off treatment not specified). No antibiotics or probiotics in last 3 months. | Faeces | 16S rRNA gene sequencing | Alpha diversity (by Chao1, Shannon, Simpson, ACE indices): significantly reduced richness (Chao1) in IBD vs HC No significant difference in richness by observed species/ACE No significant difference in diversity (Shannon, Simpson) in IBD vs HC Beta diversity (by PCA) Significant difference between IBD vs HC, active CD/UC also vs HC | **Increased in IBD vs HC** Genus level:  Escherichia, Proteobacteria **Increased in UC vs CD/HC** Phylum level:  Spirochaetes **Increased in active vs inactive CD** Phylum level:  Bacteroidetes | **Decreased in IBD vs HC** Phylum level:  Lentisphaerae **Decreased in CD vs HC** Genus level:  Haemophilus  **Decreased in UC vs HC** Genus level:  Prevotella |
| Nishino et al[97] | 2018 | Cross-sectional study | 26 | 43 | 0 | 69 | 14 | Adult | 42.0% active | Steroids, azathioprine, 5-ASA, mercaptopurine, anti-TNFa (overall patients on or off treatment not specified). No antibiotics or probiotics during study. | Mucosal brush samples (from normal mucosa adjacent to inflammation) | 16S rRNA gene sequencing | Alpha diversity (by Chao1, Shannon index) decreased significantly in IBD vs HC, no significant differences between CD vs UC Beta diversity (by PCoA) significant difference between UC vs CD vs HC; larger differences in CD | **Increased in CD vs UC/HC** Phylum level:  Proteobacteria **Increased in CD vs HC** Genus level:  Escherichia, Ruminococcus (R. gnavus), Cetobacterium, Actinobacillus, Enterococcus **Increased in UC vs HC** Genus level:  Blautia, Veillonella, Bifidobacterium, Citrobacter, Lactobacillus | **Decreased in CD vs UC/HC** Phylum level:  Firmicutes, Bacteroidetes **Decreased in CD vs UC** Phylum level:  Actinobacteria  **Decreased in CD vs HC** Genus level: Faecalibacterium, Coprococcus, Prevotella, Roseburia, Gemmiger, Alistipes, Ruminococcus (R. bromii) **Decreased in UC vs HC** Genus level:  Prevotella, Coprococcus, Pseudomonas, Alistipes |
| Imhann et al[98] | 2018 | Cross-sectional study | 188 | 107 | 18 | 313 | 582 | Adult | 25.8% active | Mesalazines, steroids, thiopurines, methotrexate, anti-TNFa (overall patients on or off treatment not specified). | Faeces | 16S rRNA gene sequencing | Alpha diversity (by Shannon index) significantly reduced in IBD vs HC Beta diversity (by PCoA) significantly different between IBD vs HC | **Increased in CD vs HC** Phylum level:  Bacteroidetes, Proteobacteria Order level:  Bacteroidales Family level:  Porphyromonadaceae, Enterobacteriaceae  Genus level:  Parabacteroides  **Increased in UC vs HC** Phylum level:  Bacteroidetes, Proteobacteria Order level:  Bacteroidales Family level:  Rikenellaceae  Genus level:  Bacteroides, Lachnobacterium, Roseburia | **Decreased in CD vs HC** Phylum level:  Actinobacteria, Tenericutes, Firmicutes Family level:  Mogibacteriaceae, Christensenellaceae, Clostridiaceae, Dehalobacteriaceae, Peptococcaceae, Peptostreptococcaceae, Ruminococcaceae Genus level:  Bifidobacterium, Ruminococcus, Faecalibacterium **Decreased in UC vs HC** Phylum level:  Firmicutes Family level:  Ruminococcaceae |
| Bajer et al[99] | 2017 | Cross-sectional study | 0 | 32 | 0 | 32 | 31 | Adult | Not specified | Steroids, azathioprine, 5-ASA, anti-TNFa (overall patients on or off treatment not specified). No antibiotics or probiotics in last 3 months. | Faeces | 16S rRNA gene sequencing | Alpha diversity (by Shannon Chao1 index): decreased in UC vs HC - no difference seen using Simpson index Beta diversity (by PCoA): significant difference in UC vs HC | **Increased in UC vs HC** Order level: Fusobacteriales Family level: Fusobacteriaceae | **Decreased in UC vs HC** Phylum level:  Verrucomicrobia Family level:  Verrucomicrobiaceae Genus level:  Coprococcus, Phascolarctobacterium, Akkermansia, Roseburia Species level:  Faecalibacterium prausnitzi, Coprococcus catus, Ruminococcus gnavus, Akkermansia muciniphila, Butyricicoccus pullicaecorum, Clostridium colinum |
| Knoll et al[100] | 2017 | Case-control study | 6 | 6 | 0 | 12 | 12 | Paediatric | 66.7% active | Mesalazines, steroids, azathioprine, methotrexate, anti-TNFa, TGF-beta2, ursodeoxycolic acid, colchicine (overall patients on or off treatment not specified). No antibiotics in last 2 months. | Faeces | Metagenomic shotgun sequencing | Alpha diversity (by Shannon index) significantly reduced in UC vs HC (by richness) significantly reduced in IBD overall vs HC Beta diversity not assessed | **Increased in IBD vs HC** Species level:  Escherichia coli, Fusobacterium nucleatum **Increased in UC vs HC** Species level:  Clostridium ramosum, Ruminococcus gnavus | **Decreased in UC vs HC** Species level:  Eubacterium rectale, Faecalibacterium prausnitzii, Bilophila wadsworthia  No CD vs HC changes |
| Sokol et al[101] | 2017 | Cross-sectional study | 149 | 86 | 0 | 235 | 38 | Adult | 45.1% active | 5-ASA, Steroids, Azathioprine, Methotrexate, Anti-TNFa (overall patients on or off treatment not specified). No antibiotics in last 3 months. | Faeces | 16S rRNA gene sequencing | Alpha diversity (by 4 indexes, not specified) significantly reduced in IBD vs HC, particularly active IBD vs HC  Beta diversity (by PCoA) significantly different between CD vs UC vs HC | **Increased in IBD vs HC** Species level:  Streptococcus anginosus, Aggregatibacter segnis | **Decreased in IBD vs HC** Genus level:  Ruminococcus, Coprococcus, Blautia, Eubacterium, Dorea, Anaerostipes, Roseburia, Faecalibacterium |
| Rehman et al[102] | 2016 | Cross-sectional study | 28 | 30 | 0 | 58 | 30 | Adult | 0% active All in remission | 5-ASA, corticosteroids, TNFa inhibitor, azathioprine, methrotrexate (overall patients on or off treatment not specified). Some patients on antibiotics during study. | Mucosal biopsies (sigmoid colon) | 16S rRNA gene sequencing | Alpha diversity (by Shannon, Chao1 index): significantly reduced in CD more than UC compared to HC Beta diversity (by PCA) differences in IBD vs HC Alpha/Beta diversity increases with age | **Increased in UC vs CD/HC** Genus level:  Bacteroides | **Decreased in IBD vs HC** Genus level:  Anaerococcus, Papillibacter **Reduced in CD vs HC** Family level:  Lachnospiraceae Genus level:  Faecalibacterium, Bacteroides, Blautia, Ruminococcus, Roseburia, Coprococcus |
| Yao et al[103] | 2016 | Case-control study | 0 | 60 | 0 | 60 | 60 | Adult | 53.3% active | No IBD medications during study No antibiotics or probiotics in last 4 weeks. | Faeces | qPCR | Not assessed | **Increased in UC vs HC** Genus level: Bacteroides | **Decreased in UC vs HC** Genus level: Clostridium Species level: Bifidobacterium species, Faecalibacterium prausnitzii |
| Hoarau et al[104] | 2016 | Cross-sectional study | 20 | 0 | 0 | 20 | 28 | Adult | 15% active | Not specified | Faeces | 16S rRNA gene sequencing | Alpha diversity (by Shannon index) - no comment on significant findings; richness in microbiome increased in CD vs relatives vs controls whereas inverse order with mycobiome Beta diversity (by PCA) - significant difference in profile of CD vs non-CD relatives vs non related controls | **Increased in CD vs related control** Species level:  Escherichia coli, Serratia marcescens, Ruminococcus gnavus | **Decreased in CD vs related control** Phylum level:  Bacteroidetes  Species level:  Faecalibacterium prausnitzii |
| Shaw et al[105] | 2016 | Cohort study | 15 | 4 | 0 | 19 | 10 | Paediatric | Not specified | All treatment-naïve | Faeces | 16S rRNA gene sequencing | Alpha diversity (by Shannon index): significantly reduced in IBD vs HC Beta diversity (by Unifrac distance calculations): differences in IBD vs HC | **Increased in IBD vs HC** Genus:  Fusobacterium, Veillonella | **Decreased in IBD vs HC** Genus level:  Coprococcus, Adlercreutzia |
| Eun et al[106] | 2016 | Cross-sectional study | 35 | 0 | 0 | 35 | 15 | Adult | 42.9% active | Prednisolone, 5-ASA, Azathioprine, Infliximab (overall patients on or off treatment not specified). No antibiotics in last 3 months. | Faeces, mucosal biopsy (ileocaecal valve) | 16S rRNA gene sequencing | Alpha diversity (by Jackknife estimator, Simpson, Shannon index): significantly reduced in CD vs HC (for faeces but not intestinal mucosa) Beta diversity (by PCoA): significantly different in CD vs HC (for faeces but not intestinal mucosa) | **Increased in CD vs HC** Class level: Gammaproteobacteria (biopsy, faeces) Bacilli, Erysipelotrichia (faeces) Family level: Enterobacteriaceae (biopsy, faeces) Fusobacteriaceae (biopsy) Pseudomonadaceae, Streptococcaceae, Erysipelotrichaceae (faeces) | **Decreased in CD vs HC** Class level: Bacteroidia, Clostridia, Negativicutes (biopsy, faeces) Family level: Bacteroidaceae, Prevotellaceae, Lachnospiraceae, Ruminococcaceae, Veillonellaceae (biopsy, faeces) |
| Naftali et al[107] | 2016 | Cross-sectional study | 31 | 0 | 0 | 31 | 5 | Adult | 35.5% active | 67.7% (21/31) patients on treatment (thiopurines, 5-ASA, corticosteroids, methotrexate). 6.5% (2/31) treatment-naïve. No antibiotics in last 1 month. | Mucosal biopsies (terminal ileum colon - healthy and inflamed tissue) | 16S rRNA gene sequencing | Alpha diversity (by Shannon index): no significant difference in ileal vs colonic CD, but generally reduced diversity in IBD vs HC Beta diversity (by PCoA): significant differences found between ileal and colonic CD | No differences to report | **Decreased in CD vs HC** Genus level: Faecalibacterium |
| Kolho et al[108] | 2015 | Cohort study | 36 | 26 | 6 | 68 | 26 | Paediatric | 30.9% active | 8.9% (6/68) on no treatment. Remaining on 5-ASA, azathioprime, methotrexate, corticosteroid, anti-TNFa inhibitor.  Some patients on antibiotics during study. | Faeces | Phylogenetic microarray, qPCR | Alpha diversity (by inverse Simpson index): reduced in IBD vs HC (decreased richness and abundance, especially butyrate producers) Beta diversity (by PCoA): significant differences noted at baseline  With anti TNF - diversity increased in those who clinically responded, with similarity to controls and beta diversity/distance measured improving | **Increased in CD vs UC/HC** Species level:  Bacteroides fragilis  **Increased in UC vs CD/HC** Species level:  Sutterella wadsworthia **Increased in CD vs UC** Species level:  Clostridium ramosum, Eubacterium cylindroides **Increased in UC vs HC** Species level:  Clostridium difficile | **Decreased in IBD vs HC** Family level:  Ruminococcae, Lachnospiraceae Genus level:  Clostridium **Decreased in CD vs HC** Species level:  Faecalibacterium prauznitzii |
| Quince et al[109] | 2015 | Cohort study | 23 | 0 | 0 | 23 | 21 | Paediatric | 100% active | Aminosalicylates, azathioprine, corticosteroids, methotrexate, biologics (overall patients on or off treatment not specified). No antibiotics in last 3 months. | Faeces | 16S rRNA gene sequencing, Shotgun metagenome sequencing | Alpha diversity (by Shannon index, OTU) significantly reduced in CD vs HC Beta diversity (by PCoA) significantly different between CD vs HC [Before EEN] | **Increased in CD vs HC** Genus level:  Atopobium (phylum Actinobacteria), Escherichia–Shigella (phylum Proteobacteria) | **Decreased in CD vs HC** Genus level:  Coprococcus, Pseudobutyrivibrio, Ruminococcus (family Lachnospiraceae) Subdoligranum, Faecalibacterium (family Clostridiales) |
| Maukonen et al[110] | 2015 | Cohort study | 10 | 12 | 0 | 22 | 8 | Paediatric | Not specified | 36.4% (8/22) treatment-naïve, others on maintenance therapy. No antibiotics in last 2 months. | Faeces | Culture, qPCR | Not assessed | **Increased in UC vs CD/HC** Genus level:  Bacteroides | **Decreased in IBD vs HC** Family level:  Lachnospiraceae, Coriobacteriaceae, Ruminococcaceae Genus level:  Bifidobacteria **Decreased in UC vs HC** Genus level:  Lactobacillus |
| Kabeerdoss et al[111] | 2015 | Cross-sectional study | 28 | 32 | 0 | 60 | 30 | Adult | Not specified | Mesalazine, sulphasalazine, purine analogue, corticosteroids (overall patients on or off treatment not specified). No antibiotics during study. | Mucosal biopsy (colon - inflamed regions) | 16S rRNA gene sequencing, qPCR | Not assessed | **Increased in IBD vs HC** Phylum level:  Bacteroidetes **Increased in UC vs CD/HC** Genus level:  Bacteroides, Prevotella, Porphyromonas, Lactobacillus Species level: Escherichia coli **Increased in CD vs UC/HC** Phylum level:  Proteobacteria | **Decreased in CD vs HC** Genus level:  Clostridium coccoides, Eubacterium rectale, Firmicutes **Decreased in CD vs UC** Genus level:  Clostridium leptum Species level:  Faecalibacterium prausnitzii |
| Chen et al[112] | 2014 | Cross-sectional study | 26 | 46 | 0 | 72 | 21 | Adult | 83% active | IBD medications not specified. No antibiotics or probiotics in last 2 months. | Faeces, mucosal biopsies (terminal ileum, caecum, rectum) | 16S rRNA gene sequencing | Alpha diversity (by Shannon, Chao1 index): significantly reduced in IBD vs HC Beta diversity (by PCA): significantly different between IBD vs HC, no significant differences in CD vs UC | **Increased in IBD vs HC** Phylum level:  Proteobacteria Family level:  Streptococceae, Enterococcaceae Genus level:  Escherichia-Shigella,  Enterococcus **Increased in CD vs HC** Phylum:  Fusobacteria **Increased in UC vs HC** Phylum:  Bacteroides | **Decreased in IBD vs HC** Family level:  Lachnospiraceae Genus level:  Roseburia, Coprococcus, Ruminococcus **Decreased in CD vs HC** Genus level:  Faecalibacterium |
| Gevers et al[113] | 2014 | Cohort study | 447 | 0 | 0 | 447 | 221 | Paediatric | Not specified | Treatment-naïve. 13% (57/447) on antibiotics during sample collection. | Faeces, mucosal biopsies (terminal ileum, rectum) | 16S rRNA gene sequencing | Not assessed | **Increased in CD vs HC** Family level: Enterobacteriaceae, Pasteurellacaea, Veillonellaceae, Fusobacteriaceae | **Decreased in CD vs HC** Phylum level:  Bacteroides  Genus:  Faecalibacterium, Roseburia, Blautia, Ruminococcus, Coprococcus Order level:  Clostridiales   Family level:  Lachnospiraceae, Erysipelotrichaceae |
| Said et al[114] | 2014 | Cross-sectional study | 21 | 14 | 0 | 35 | 24 | Adult | 54% active | Anti-inflammatories, immunosuppressors, anti-TNFa inhibitor (overall patients on or off treatment not specified). | Saliva | 16S rRNA gene sequencing | Alpha diversity (by Shannon, Simpson, Fisher alpha, Chao1, ACE index): no significant difference in IBD vs HC Beta diversity (by PCoA): significantly different between IBD (CD/UC) vs HC | **Increased in CD/UC vs HC** Phylum level: Bacteroidetes Genus level: Prevotella, Veillonella | **Decreased in CD/UC vs HC** Phylum level:  Proteobacteria Genus level:  Streptococcus, Haemophilus **Decreased in CD vs HC** Genus level:  Neisseria, Gemella |
| Tong et al[115] | 2013 | Cross-sectional study | 16 | 16 | 0 | 32 | 32 | Adult | Not specified | Not specified | Muosal biopsies (descending colon, rectum) | 16S rRNA gene sequencing | Alpha diversity (through OTU levels): reduced in IBD vs HC; significantly more so in CD vs UC Beta diversity (through PCoA): significantly different in IBD vs HC, more so CD vs UC | **Increased in** **IBD vs HC** Phylum level:  Actinobacteria | **Decreased in IBD vs HC** Phylum level: Firmicutes |
| Prideaux et al[116] | 2013 | Cross-sectional study | 22 | 30 | 0 | 52 | 29 | Adult | Not specified | Mesalazine, thiopurine, anti-TNFa inhibitor (overall patients on or off treatment not specified). No antibiotics or probiotics in last 1 month. | Mucosal biopsies (terminal ileum, caecum, rectum) | 16S rRNA gene sequencing | Alpha diversity (by Shannon index): significantly reduced in CD vs HC Beta diversity (by PCA): significantly different between IBD vs HC | **Increased in CD vs HC** Species level: Enterococcus faecium **Increased in UC vs HC** Species level:  Spirochaeta halophila, Leptospira parva, Nocardia farcinica, Chlamydophila pneumonia | **Decreased in CD/UC vs HC** Genus level:  Coprococcus, Dorea **Decreased in CD vs HC** Phylum level:  Bacteroidetes Family level:  Ruminococcaceae Genus level:  Faecalibacterium, Roseburia, Lachnospiraceae, Clostridiales **Decreased in UC vs HC** Phylum level:  Firmicutes Species level:  Runella slithyformis, Mycoplasma bovis, Cellulomonas flavigena, Tannerella forsythensis, Rhodothermus marinus |
| Pérez-Brocal et al[117] | 2013 | Cross-sectional study | 11 | 0 | 0 | 11 | 8 | Adult | 90% active | Corticosteroids, azathioprine, anti-TNFa therapy (overall patients on or off treatment not specified). No antibiotics in last 3 months. | Faeces | 16S rRNA gene sequencing | Alpha diversity (by Chao1, Shannon index) significantly reduced in CD vs HC Beta diversity (by PCoA) significantly different between CD vs HC | **Increased in CD vs HC** Phylum level: Proteobacteria Genus level: Veillonella (order Clostridiales) Species level: Escherichia coli (family Enterobacteriaceae), Clostridium bolteae (order Clostridiales) | **Decreased in CD vs HC**  Phylum level:  Firmicutes, Temerocites Class level: Clostridia Order level: Bacteroidales Species level: Collinsella aerofaciens |
| Docktor et al[23] | 2012 | Cross-sectional study | 40 | 31 | 0 | 71 | 43 | Paediatric | 32% active | 50.6% (42/83) on immunosuppressive medications (methotrexate, 6-mercaptupurine, azathioprine, tacrolimus, cyclosporine, anti-TNFa inhibitor) No antibiotics in last 4 weeks | Tongue/buccal swab | 16S rRNA gene sequencing | Alpha diversity (by Shannon index) significantly reduced in CD vs HC  - not reduced in UC vs HC Beta diversity (by PCA) not significantly different between CD vs HC | **Increased in CD vs HC** Phylum level:  Spirochaetota, Synergistetes, Bacteroidetes | **Decreased in CD vs HC** Phylum level:  Firmicutes, Fusobacteria |

***Table S2***: Newcastle-Ottawa Scale for Cohort Studies. Each star represents a point awarded in the category.[20]

|  | | **Cohort Studies - Selection** | | | | **Comparability** | | **Cohort Study - Outcome** | | | **TOTAL SCORES** |
| --- | --- | --- | --- | --- | --- | --- | --- | --- | --- | --- | --- |
| **Author** | **Year of Publication** | Representativeness | Selection of non exposed cohort | Ascertaining exposure | Outcome not present at start | Primary study control: age | Additional factors: antibiotics | Assessment of outcome | Long enough follow-up | Adequacy of follow-up |  |
| Alahdal et al[43] | 2024 | ★ | - | ★ | ★ | ★ | ★ | ★ | ★ | - | 7 |
| Elmaghrawy et al[48] | 2023 | ★ | - | ★ | ★ | ★ | - | ★ | ★ | - | 6 |
| Park et al[62] | 2022 | ★ | ★ | ★ | ★ | ★ | ★ | ★ | ★ | - | 8 |
| Frau et al[72] | 2021 | ★ | ★ | ★ | ★ | - | ★ | ★ | ★ | - | 7 |
| Nishihara et al[74] | 2021 | ★ | ★ | ★ | ★ | ★ | ★ | ★ | ★ | - | 8 |
| Sanchis-Artero et al[81] | 2021 | ★ | ★ | ★ | ★ | ★ | ★ | ★ | ★ | - | 8 |
| Somineni et al[82] | 2021 | ★ | - | ★ | ★ | ★ | ★ | ★ | ★ | - | 7 |
| Kansal et al[90] | 2019 | ★ | ★ | ★ | ★ | ★ | ★ | ★ | ★ | - | 8 |
| de Meij et al[94] | 2018 | ★ | ★ | ★ | ★ | - | ★ | ★ | ★ | - | 7 |
| Walujkar et al[95] | 2018 | ★ | ★ | ★ | ★ | ★ | ★ | ★ | ★ | - | 8 |
| Shaw et al[105] | 2016 | ★ | ★ | ★ | ★ | - | - | ★ | ★ | - | 6 |
| Kolho et al[108] | 2015 | ★ | - | ★ | ★ | - | - | ★ | ★ | - | 5 |
| Quince et al[109] | 2015 | ★ | ★ | - | ★ | - | ★ | ★ | ★ | - | 6 |
| Maukonen et al[110] | 2015 | ★ | - | ★ | ★ | - | ★ | ★ | ★ | - | 6 |
| Gevers et al[113] | 2014 | ★ | - | - | ★ | ★ | - | ★ | ★ | - | 5 |

***Table S3***: Newcastle-Ottawa Scale for Case-Control Studies. Each star represents a point awarded in the category.[20]

|  | | **Case-control study - Selection** | | | | **Comparability** | | **Case-control study - Exposure** | | | **TOTAL SCORES** |
| --- | --- | --- | --- | --- | --- | --- | --- | --- | --- | --- | --- |
| **Author** | **Year of Publication** | Case definition | Representativeness | Selection of Controls | Definition of Controls | Primary study control: age | Additional factors: antibiotics | Ascertaining exposure | Same method ascertaining for case & controls | Equal non response rate |  |
| Scanu et al[42] | 2024 | ★ | ★ | - | ★ | ★ | ★ | ★ | ★ | ★ | 8 |
| Han et al[44] | 2024 | ★ | ★ | ★ | ★ | ★ | ★ | ★ | ★ | ★ | 9 |
| Räisänen et al[51] | 2023 | ★ | ★ | ★ | - | ★ | ★ | ★ | ★ | - | 7 |
| Wang et al[67] | 2022 | ★ | ★ | - | ★ | ★ | ★ | ★ | ★ | ★ | 8 |
| Hu et al[68] | 2021 | ★ | ★ | - | ★ | ★ | ★ | ★ | ★ | ★ | 8 |
| Tang et al[69] | 2021 | ★ | ★ | - | - | - | ★ | ★ | ★ | ★ | 6 |
| Olbjørn et al[89] | 2019 | ★ | ★ | ★ | ★ | - | ★ | ★ | ★ | ★ | 8 |
| Xun et al[93] | 2018 | ★ | ★ | - | ★ | ★ | ★ | ★ | ★ | ★ | 8 |
| Imhann et al[98] | 2018 | ★ | ★ | ★ | ★ | ★ | - | ★ | ★ | ★ | 8 |
| Knoll et al[100] | 2017 | ★ | ★ | ★ | ★ | ★ | ★ | ★ | ★ | ★ | 9 |
| Yao et al[103] | 2016 | ★ | ★ | - | ★ | ★ | ★ | ★ | ★ | ★ | 8 |

***Table S4***: Adapted Newcastle-Ottawa Scale for Cross-Sectional Studies. Each star represents a point awarded in the category.[20, 21]

|  | | | **Cross-sectional study - Selection** | | | | | **Comparability** | | | **Cross-sectional study - Outcome** | | **TOTAL SCORES** |
| --- | --- | --- | --- | --- | --- | --- | --- | --- | --- | --- | --- | --- | --- |
| **Author** | **Year of Publication** | Representativeness | | Sample Size | Non-response rate | Ascertaining exposure screening tool (2) | Primary study control: age | | Additional factors: antibiotics | Assessment of outcome (2) | | Statistical test |  |
| Budzinski et al[38] | 2025 | ★ | | - | ★ | ★★ | ★ | | - | ★★ | | ★ | 8 |
| Zheng et al[39] | 2024 | ★ | | - | ★ | ★★ | ★ | | ★ | ★★ | | ★ | 9 |
| Chen et al[40] | 2024 | ★ | | - | ★ | ★★ | ★ | | - | ★★ | | ★ | 8 |
| Conrad et al[41] | 2024 | ★ | | - | ★ | ★★ | ★ | | ★ | ★★ | | ★ | 9 |
| Alsulaiman et al[45] | 2023 | ★ | | - | ★ | ★★ | ★ | | ★ | ★★ | | ★ | 9 |
| Markelova et al[46] | 2023 | ★ | | - | ★ | ★★ | - | | ★ | ★★ | | ★ | 8 |
| Lopez et al[47] | 2023 | ★ | | - | ★ | ★★ | - | | ★ | ★★ | | ★ | 8 |
| Wu et al[49] | 2023 | ★ | | - | ★ | ★★ | - | | - | ★★ | | ★ | 7 |
| Al-Amrah et al[50] | 2023 | ★ | | - | ★ | ★★ | - | | ★ | ★★ | | ★ | 8 |
| Gao et al[52] | 2023 | ★ | | - | ★ | ★★ | - | | - | ★★ | | - | 6 |
| Salimi et al[53] | 2022 | - | | - | ★ | ★★ | - | | ★ | ★★ | | ★ | 7 |
| Zhu et al[54] | 2022 | ★ | | ★ | ★ | ★★ | ★ | | ★ | ★★ | | ★ | 10 |
| Xu et al[55] | 2022 | ★ | | - | ★ | ★★ | ★ | | - | ★★ | | ★ | 8 |
| Jun Hu et al[56] | 2022 | ★ | | - | ★ | ★★ | ★ | | - | ★★ | | ★ | 8 |
| Ma et al[30] | 2022 | ★ | | - | ★ | ★★ | ★ | | ★ | ★★ | | ★ | 9 |
| Jacobs et al[57] | 2022 | ★ | | - | ★ | ★★ | - | | - | ★★ | | ★ | 7 |
| Sternes et al[58] | 2022 | ★ | | - | ★ | ★★ | - | | ★ | ★★ | | ★ | 8 |
| Paljetak et al[59] | 2022 | ★ | | - | ★ | ★★ | - | | ★ | ★★ | | ★ | 8 |
| Hu et al[60] | 2022 | ★ | | - | ★ | ★★ | ★ | | ★ | ★★ | | ★ | 9 |
| Zuo et al[61] | 2022 | ★ | | - | ★ | ★★ | ★ | | ★ | ★★ | | ★ | 9 |
| Berbisá et al[22] | 2022 | ★ | | - | ★ | ★★ | ★ | | ★ | ★★ | | ★ | 9 |
| Barberio et al[63] | 2022 | ★ | | - | ★ | ★★ | - | | ★ | ★★ | | ★ | 8 |
| Sukhina et al[64] | 2022 | ★ | | ★ | ★ | - | - | | - | ★ | | ★ | 5 |
| Teofani et al[65] | 2022 | ★ | | - | ★ | ★★ | ★ | | ★ | ★★ | | ★ | 9 |
| Wan et al[66] | 2022 | ★ | | - | ★ | ★★ | ★ | | ★ | ★★ | | ★ | 9 |
| Ostrowski et al[70] | 2021 | ★ | | - | ★ | ★★ | - | | ★ | ★★ | | ★ | 8 |
| Liu et al[71] | 2021 | ★ | | - | ★ | ★★ | ★ | | ★ | ★★ | | ★ | 9 |
| Liang et al[73] | 2021 | ★ | | - | ★ | ★★ | - | | - | ★★ | | ★ | 7 |
| Xia et al[75] | 2021 | ★ | | - | ★ | ★★ | ★ | | - | ★★ | | ★ | 8 |
| Maldonado-Arriaga et al[76] | 2021 | ★ | | - | ★ | ★★ | ★ | | ★ | ★★ | | ★ | 9 |
| Dai et al[77] | 2021 | ★ | | ★ | ★ | ★★ | - | | ★ | ★★ | | ★ | 9 |
| Chang et al[78] | 2021 | ★ | | - | ★ | ★★ | ★ | | ★ | ★★ | | ★ | 9 |
| Abdul-Hussein et al[79] | 2021 | ★ | | - | ★ | ★★ | - | | - | ★★ | | ★ | 7 |
| Juyal et al[80] | 2021 | ★ | | ★ | ★ | ★★ | - | | ★ | ★★ | | ★ | 9 |
| Alam et al[83] | 2020 | - | | - | - | ★★ | - | | - | ★★ | | ★ | 5 |
| Sila et al[84] | 2020 | ★ | | - | ★ | ★★ | - | | - | ★★ | | ★ | 7 |
| Qiu et al [85] | 2020 | ★ | | - | ★ | ★★ | - | | ★ | ★★ | | ★ | 8 |
| Kowalska-Duplaga et al[86] | 2019 | ★ | | - | ★ | ★★ | ★ | | ★ | ★★ | | ★ | 9 |
| Guo et al[87] | 2019 | ★ | | - | ★ | ★★ | - | | ★ | ★★ | | ★ | 8 |
| Malham et al[88] | 2019 | ★ | | - | ★ | ★★ | ★ | | ★ | ★★ | | ★ | 9 |
| Zhong et al[91] | 2019 | ★ | | - | ★ | ★★ | ★ | | ★ | ★★ | | ★ | 9 |
| Al-Bayati et al[92] | 2018 | ★ | | ★ | ★ | ★★ | ★ | | ★ | ★★ | | ★ | 10 |
| Ma et al[96] | 2018 | ★ | | - | ★ | ★★ | ★ | | ★ | ★★ | | ★ | 9 |
| Nishino et al[97] | 2018 | ★ | | - | ★ | ★★ | ★ | | ★ | ★★ | | ★ | 9 |
| Bajer et al[99] | 2017 | ★ | | - | ★ | ★★ | ★ | | - | ★★ | | ★ | 8 |
| Sokol et al[101] | 2017 | ★ | | ★ | ★ | ★★ | ★ | | ★ | ★★ | | ★ | 10 |
| Rehman et al[102] | 2016 | ★ | | - | ★ | ★★ | - | | - | ★★ | | ★ | 7 |
| Hoarau et al[104] | 2016 | ★ | | - | ★ | ★★ | ★ | | - | ★★ | | ★ | 8 |
| Eun et al[106] | 2016 | ★ | | - | ★ | ★★ | ★ | | ★ | ★★ | | ★ | 9 |
| Naftali et al[107] | 2016 | ★ | | - | ★ | ★★ | - | | ★ | ★★ | | ★ | 8 |
| Kabeerdoss et al[111] | 2015 | ★ | | - | ★ | ★★ | ★ | | - | ★★ | | ★ | 8 |
| Chen et al[112] | 2014 | ★ | | - | ★ | ★★ | - | | ★ | ★★ | | ★ | 8 |
| Said et al[114] | 2014 | ★ | | - | ★ | ★★ | - | | - | ★★ | | ★ | 7 |
| Tong et al[115] | 2013 | ★ | | ★ | ★ | ★ | ★ | | - | ★★ | | ★ | 8 |
| Prideaux et al[116] | 2013 | ★ | | - | ★ | ★★ | ★ | | ★ | ★★ | | ★ | 9 |
| Pérez-Brocal et al[117] | 2013 | ★ | | - | ★ | - | - | | ★ | ★★ | | ★ | 6 |
| Docktor et al[23] | 2012 | ★ | | - | ★ | ★★ | ★ | | ★ | ★★ | | - | 8 |
